# A radiomics–deep learning nomogram integrating intratumoral and peritumoral DCE-MRI features for pCR prediction in breast cancer

**DOI:** 10.3389/fonc.2026.1830219

**Published:** 2026-09-16

**Authors:** Yiru Wang, Haibo Wang, Jian Cui

**Affiliations:** Breast Disease Diagnosis and Treatment Center, The Affiliated Hospital of Qingdao University, Qingdao, China

**Keywords:** breast cancer, deep learning, intratumoral, machine learning, neoadjuvant systemic therapy, pathological complete response, peritumoral, radiomics

## Abstract

**Background:**

Pathological complete response (pCR) after neoadjuvant systemic therapy (NST) is an important surrogate endpoint for breast cancer prognosis. Reliable pretreatment prediction of pCR remains difficult, as conventional imaging underutilizes the peritumoral microenvironment, including immune infiltration, matrix remodeling, and vascular dynamics. We constructed a multimodal nomogram combining intratumoral and peritumoral dynamic contrast-enhanced magnetic resonance imaging (DCE-MRI) radiomic features, 3D deep learning (DL) features, and clinical biomarkers to predict pCR.

**Methods:**

In this single-center retrospective study, 534 patients were randomly split into training (n = 373) and internal test (n = 161) sets. Four classifiers were compared at 3-, 6-, and 9-mm peritumoral extensions. A 3D ResNet-50 model with Squeeze-and-Excitation attention was used to extract DL features from the training-set-selected TP6 region. Clinical predictors were selected by multivariate logistic regression. The nomogram was evaluated using area under the curve (AUC), calibration, and decision curve analysis (DCA).

**Results:**

The 6-mm peritumoral margin consistently yielded the numerically highest AUC across all classifiers in the training set. However, in the held-out test set, the AUC difference between Model-TP6 and the intratumoral-only model was not statistically significant (DeLong P = 0.358) and should therefore be interpreted as a numerical trend rather than a statistically confirmed advantage. The integrated nomogram achieved the numerically highest test-set AUC (0.883, 95% CI: 0.827-0.940). Although most pairwise DeLong comparisons did not reach statistical significance, it showed a favorable combination of discrimination, calibration, and decision-curve net benefit within this internal cohort.

**Conclusions:**

This nomogram synergizes radiomics with DL representations, offering an exploratory, preliminary pretreatment tool for stratifying NST candidates. However, in this retrospective single-center study, the model was developed and evaluated using an internal split without independent external validation, and its performance may therefore be optimistic and not generalizable to other institutions or imaging settings. Prospective multicenter external validation is essential before any clinical application.

## 1 Introduction

In 2025, the United States epidemiological data underscored the burden of breast cancer (1): more than 310,000 new cases and over 40,000 deaths. Breast cancer accounts for about 32% of new cancer diagnoses and 15% of cancer deaths in women, making it the most commonly diagnosed cancer in this group.

As a well-established clinical benchmark for the management of locally advanced breast cancer, NST aims to facilitate surgical resection and potential breast conservation by inducing tumor regression and clinical downstaging. Evaluation of therapeutic response relies on standardized criteria, encompassing progressive disease, stable disease, partial response, and pathological complete response (pCR). Prognostically, patients achieving pCR exhibit markedly prolonged disease-free survival and overall survival, a correlation that is particularly strong in triple-negative and HER2-enriched breast cancers (2–4). Current assessment strategies rely on clinical examination, imaging, and postoperative pathological evaluation, with histopathological examination of resected specimens serving as the gold standard. However, these methods only provide post-treatment information and have limited capacity to reflect tumor heterogeneity or dynamic biological changes during treatment.

Thus, early and accurate evaluation of NST efficacy carries great significance for optimizing subsequent treatment, guiding individualized surgical strategies, and predicting prognosis. Among traditional imaging modalities, dynamic contrast-enhanced magnetic resonance imaging (DCE-MRI) is the standard approach for evaluating treatment response and residual lesions. It allows for quantitative assessment of tumor morphology (e.g., size and volume) and hemodynamic parameters (e.g., *K^trans^* and *K_ep_*), providing valuable functional and kinetic information. Nevertheless, conventional analysis of these parameters is often performed qualitatively or semi-quantitatively in routine practice and remains subject to inter-reader variability. Moreover, routine assessment often emphasizes changes in tumor size and may incompletely capture tumor heterogeneity and microscale treatment-related changes. These limitations highlight the demand for more advanced quantitative imaging strategies.

Initially conceptualized by Lambin et al. (5) in 2012, radiomics relies on automated processing to extract a wide spectrum of quantitative features from medical images. By combining these features with machine learning, routine diagnostic images can be converted into quantitative data, allowing detailed assessment of intratumoral morphological heterogeneity. Radiomics can uncover microscopic structural and functional information invisible to the naked eye, thereby linking imaging phenotypes to genomic phenotypes (6). MRI-based radiomics has demonstrated promising applications in breast cancer, including differential diagnosis (7–9), molecular subtype classification (10, 11), prediction of lymph node metastasis (12–14), and assessment of neoadjuvant treatment response (15, 16).

Tumors do not exist as isolated entities; the tumor microenvironment (TME) plays a pivotal role in tumorigenesis, progression, and metastasis, and represents a key therapeutic target (17). The imaging heterogeneity of the peritumoral region essentially reflects microenvironmental remodeling induced by tumor invasion, including local inflammatory activation, tissue structural reorganization, and dynamic alterations in the extracellular matrix (18). These microscopic alterations provide important visual evidence for indirectly assessing tumor invasiveness and biological behavior. Consequently, capturing comprehensive information from both the intratumoral and peritumoral regions is essential for predicting NST efficacy. Although previous studies have attempted to integrate intratumoral and peritumoral features, systematic investigations into their utility for predicting NST response in breast cancer remain limited. In particular, there is a lack of in-depth analysis of the synergistic effects of these multi-regional features (19–21).

Deep learning (DL) autonomously extracts intricate tumor features via an end-to-end paradigm (6) and has shown broad utility in breast cancer imaging, including ultrasound-based detection and early diagnosis (22, 23). In breast MRI, DL provides an objective, quantitative approach for assessing NST efficacy (24–26). However, the “black-box” nature of DL limits its clinical interpretability (27, 28). To address this drawback, traditional radiomics provides explicit mathematical characterizations of tumor heterogeneity (e.g., shape and texture). Accordingly, the development of hybrid models that synergize DL with traditional radiomics has emerged as a major research focus.

Despite the widespread application of MRI radiomics, limitations persist: First, tumor boundary delineation is ambiguous, and no uniform standard has been established for selecting an appropriate peritumoral margin for pCR prediction. Second, model comparisons remain insufficient, and the relative performance, limitations, and complementarity of traditional radiomics versus deep learning in pCR prediction require systematic verification. This study first compared candidate peritumoral margins using DCE-MRI radiomics and selected one margin exclusively within the training set for subsequent modeling. Subsequently, a 3D Squeeze-and-Excitation ResNet-50 was used to extract deep learning features from the training-set-selected region and perform a head-to-head comparison with the traditional radiomics model. To realize our primary objective of constructing a candidate noninvasive prediction tool for pCR following NST in breast cancer, this study developed and internally tested a combined nomogram incorporating clinical predictors, traditional radiomics, and deep-learning features.

## 2 Materials and methods

### 2.1 Patients and data

#### 2.1.1 Pathological screening and enrollment criteria

A retrospective review was conducted on clinical and imaging data derived from patients with pathologically confirmed breast cancer (via core needle biopsy) who received preoperative NST and subsequent surgery at our institution between January 2021 and December 2023 (flowchart shown in **Figure 1**). The inclusion criteria were as follows: (1) Unilateral primary invasive breast cancer of no special type (pathologically confirmed); (2) Detailed breast MRI examination performed before core needle biopsy and prior to NST, with acceptable image quality; (3) Available baseline clinical characteristics and complete pathological data (pre- and post-treatment); and (4) No prior anti-tumor therapy received before NST. The exclusion criteria were as follows: (1) MRI images of insufficient quality for analysis; (2) Missing key clinical or pathological data; (3) A history of breast surgery, radiotherapy, or chemotherapy; (4) Bilateral breast cancer, occult breast cancer, or distant metastasis at presentation; (5) History of other malignant neoplasms; (6) Failure to receive standard neoadjuvant systemic therapy; and (7) Non-mass enhancement lesions or lesions with ill-defined margins for which accurate region of interest (ROI) delineation was not feasible.

**Figure 1:**
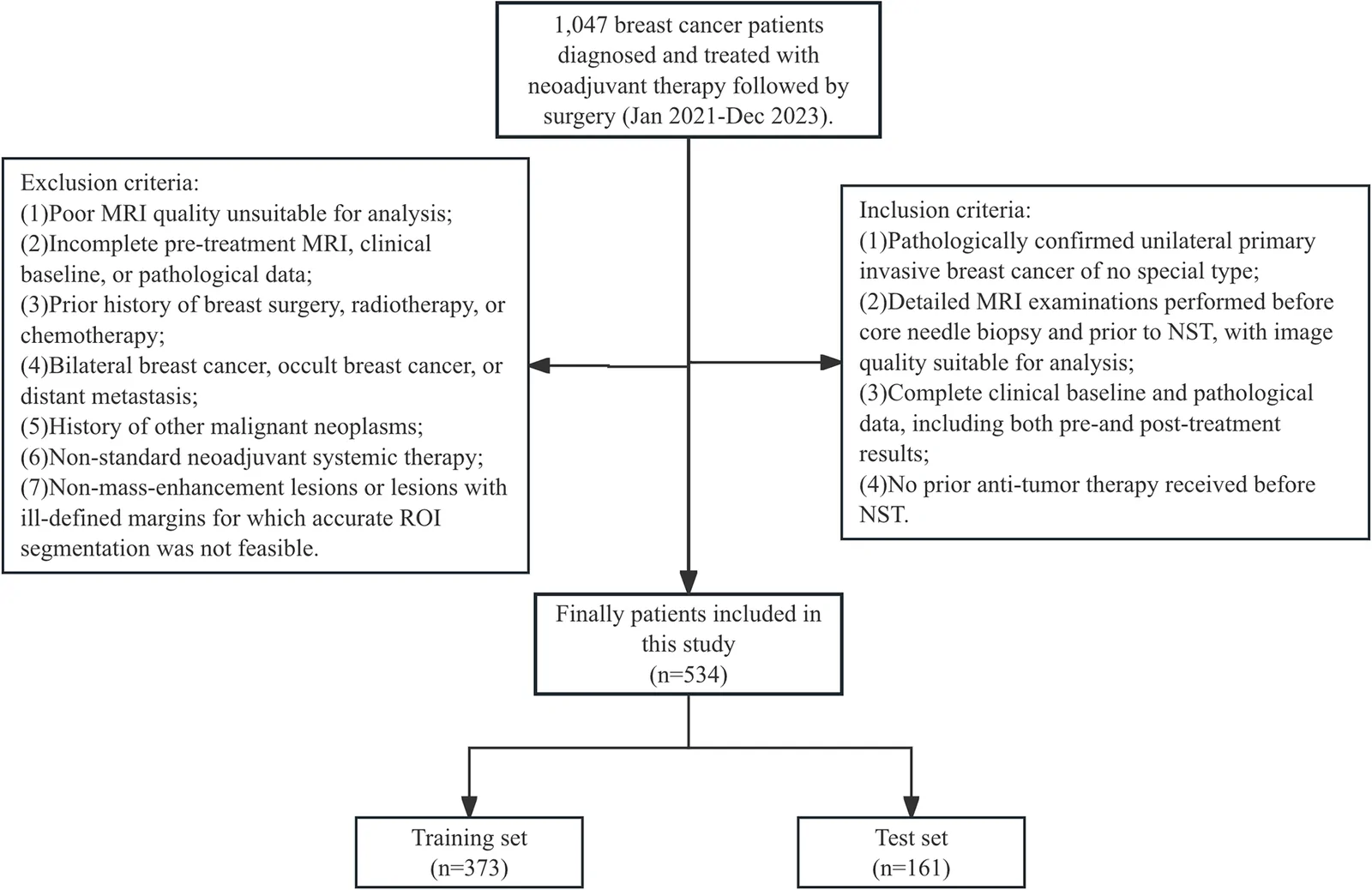
Flowchart of case inclusion, exclusion, and data grouping.

In total, 534 patients were finally enrolled. They were randomly split into the training and test sets at a 7:3 ratio. The balance of baseline characteristics between the two groups was statistically assessed.

#### 2.1.2 Data collection and interpretation criteria

Clinical and pathological features collected included age, body mass index (BMI), menstrual status, maximum dimension, tumor–node–metastasis (TNM) stage, estrogen receptor (ER), progesterone receptor (PR), HER2, Ki-67, molecular subtype, pathological grade, lymph node status, and Miller-Payne grade.

The interpretation criteria were as follows: ER and PR were considered positive if ≥1% of cells exhibited nuclear staining (29); HER2 was defined as positive if the immunohistochemistry (IHC) score was 3+ or fluorescence *in situ* hybridization (FISH) confirmed gene amplification (30); Ki-67 was classified as high expression if the value exceeded 20%. Molecular subtypes were categorized per standard guidelines into Luminal A, Luminal B, human epidermal growth factor receptor 2 (HER2)-enriched, and triple-negative breast cancer.

NST was generally administered according to molecular subtype and receptor status in accordance with clinical guidelines. Specifically, patients with hormone receptor-positive/HER2-negative (HR+/HER2−) disease patients received anthracycline-taxane sequential regimens (e.g., EC-T or AC-T, 8 cycles); HER2-positive patients received anthracycline/taxane combined with trastuzumab ± pertuzumab (e.g., TCbHP or AC-THP, 6–8 cycles); and triple-negative patients received anthracycline-taxane ± platinum (6–8 cycles) (31). Because treatment was assigned generally according to subtype and receptor status per guidelines, regimen allocation was largely determined by the receptor variables already included in the clinical model.

#### 2.1.3 Efficacy evaluation endpoint

The primary endpoint was pCR, defined as the absence of residual invasive carcinoma in the breast and sampled regional lymph nodes, irrespective of residual ductal carcinoma *in situ* (ypT_0/is_ ypN_0_). Histopathological response in the breast was additionally graded according to the Miller–Payne grading system.

### 2.2 MRI data acquisition

All included patients underwent baseline MRI within 1–2 weeks before NST initiation using a 3.0 T MRI scanner (GE Signa HDxt) with a dedicated 16-channel breast coil. All baseline MRI examinations were performed before core needle biopsy to avoid biopsy-induced changes such as hemorrhage, inflammation, or edema. As a result, the extracted radiomic features were not affected by post-biopsy changes. Patients were placed in the prone position to ensure natural suspension of the bilateral breasts. DCE-MRI comprised one unenhanced fat-suppressed T1-weighted imaging (T1WI) phase and eight enhanced phases. Gd-DTPA (0.1 mL/kg) was administered intravenously at 2 mL/s, followed by a 20 mL saline flush. The first post-contrast phase began 60 s after injection, with seven subsequent phases acquired continuously. Each phase lasted approximately 65 s. Key scanning parameters were: TR 4.25 ms, TE 2.1 ms, slice thickness 0.7 mm, slice gap 1.5 mm, matrix 320×320, and FOV 380×380 mm. All raw images were exported in DICOM format from the picture archiving and communication system (PACS). Image quality was rigorously assessed by two experienced radiologists; cases with significant motion artifacts, image distortion, or failure to meet the radiomics analysis criteria were excluded.

### 2.3 Definition and segmentation of ROI

#### 2.3.1 Image preprocessing

To ensure robust feature extraction, we analyzed the early enhanced phase (Phase 1) of DCE-MRI, which corresponds to the most prominent lesion enhancement. Before segmentation, raw images were processed using a standardized preprocessing pipeline. The image preprocessing pipeline commenced with N4 bias correction to compensate for magnetic field non-uniformities. We subsequently resolved spatial inconsistencies by resampling the volumetric data to a standard 1×1×1 mm³ voxel grid. Finally, Z-score normalization was applied to standardize grayscale intensity distributions, thus improving comparability of signal-intensity distributions across patients.

#### 2.3.2 Intratumoral region segmentation

Tumor segmentation was meticulously executed in a double-blinded manner by a pair of experienced radiologists, each possessing over a decade of clinical expertise. Utilizing the ITK-SNAP platform (version 4.2), they manually traced the neoplastic margins on a slice-by-slice basis. In instances where patients presented with multifocal disease, the analysis strictly targeted only the single lesion demonstrating the maximum diameter. A senior specialist with 20 years of clinical experience reviewed all preliminary segmentations. The final 3D volume of interest (VOI) was established only after this review. To assess the repeatability of segmentation, two radiologists independently segmented a randomly selected subset of 50 cases from the training set. A Dice similarity coefficient (DSC) > 0.85 indicated good spatial consistency. The intraclass correlation coefficient (ICC) was then calculated in the training set to evaluate the robustness of radiomic features against segmentation variability. Only robust features with ICC > 0.75 were retained for subsequent feature selection and modeling. This design ensured that repeatability-based feature screening was confined to the training set, thereby avoiding information leakage to the test set.

#### 2.3.3 Definition and refinement of the peritumoral region

We used Python 3.7 to automatically generate 3 mm, 6 mm, and 9 mm peritumoral regions (P3, P6, and P9, respectively) outside the tumor margin by expanding the manually segmented tumor ROI. A 3 mm step size was chosen to balance spatial resolution, computational cost, and the burden of multiple comparisons. All peritumoral ROIs were manually inspected to exclude skin, nipples, chest wall muscles, and non-glandular tissue outside the breast parenchyma. This process ensured the accuracy of subsequent feature extraction. The schematic illustration is depicted in **Figure 2**.

**Figure 2:**
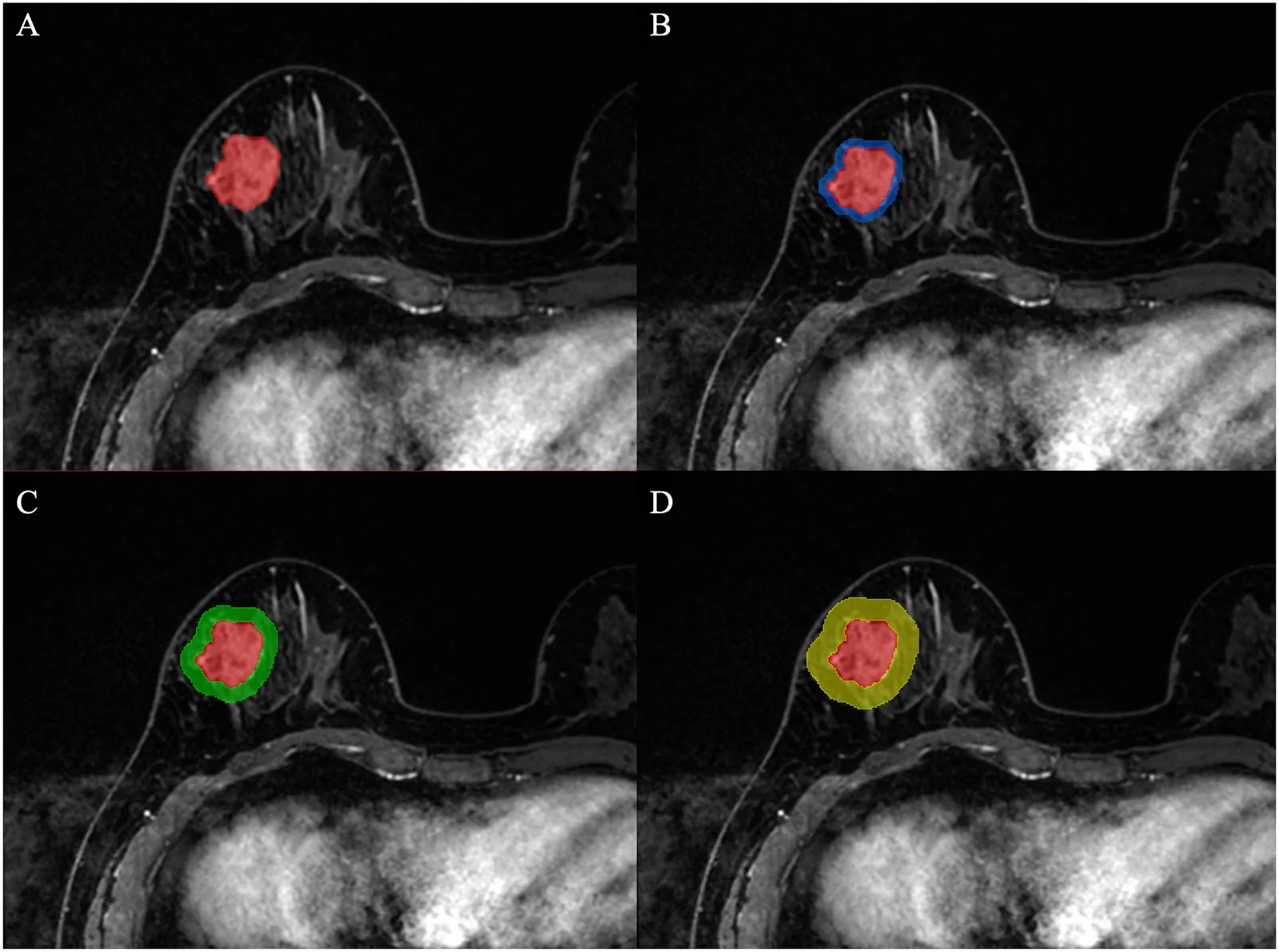
Schematic of intratumoral and peritumoral ROI segmentation. **(A)** Intratumoral region; **(B)** Intratumoral plus 3-mm peritumoral region; **(C)** Intratumoral plus 6-mm peritumoral region; **(D)** Intratumoral plus 9-mm peritumoral region.

### 2.4 Radiomics feature extraction

#### 2.4.1 Traditional radiomics feature extraction

High-throughput features were extracted separately from intratumoral and peritumoral ROIs using PyRadiomics 3.1, based on original images and images processed with Wavelet transformation and Laplacian of Gaussian (LoG) filtering (σ = 2.0, 3.0, 4.0, and 5.0 mm). The feature classes comprised shape features, first-order statistics, and texture features derived from the Gray Level Cooccurrence Matrix (GLCM), Gray Level Dependence Matrix (GLDM), Gray Level Run Length Matrix (GLRLM), Gray Level Size Zone Matrix (GLSZM), and Neighborhood Gray Tone Difference Matrix (NGTDM). In this study, four regional feature configurations were constructed:

1. T: Intratumoral features only;
2. TP3: Intratumoral features combined with features from the 3-mm peritumoral ROI;
3. TP6: Intratumoral features combined with features from the 6-mm peritumoral ROI;
4. TP9: Intratumoral features combined with features from the 9-mm peritumoral ROI.

#### 2.4.2 Deep learning-based feature extraction

DL features were extracted from the training-set-selected TP6 region using a 3D Squeeze-and-Excitation ResNet-50 architecture. To reduce overfitting on our moderately sized dataset, the model was initialized with MedicalNet pre-trained weights and fine-tuned on the training set only. The test set was strictly held out and was never used for architecture design, training, or hyperparameter tuning. All input volumes were standardized to 72×72×72 voxels. To ensure rigorous reproducibility and robust training, several key implementation strategies were adopted. First, the training set was internally partitioned using stratified 5-fold cross-validation. Online data augmentation (including mild rotation, translation, scaling, intensity perturbation, and Gaussian noise) was applied solely to the training folds. Second, to address class imbalance, the network was optimized using a class-weighted binary cross-entropy loss via a stochastic gradient descent (SGD) optimizer (learning rate = 0.001, batch size = 96). Third, training was capped at a maximum of 50 epochs, employing early stopping (patience = 10 epochs based on validation loss) to retain the optimal weights. To guarantee reproducibility, a fixed random seed (0) was applied to all stochastic procedures. All deep learning experiments were executed in Python 3.7.12 utilizing PyTorch 1.13.0 and CUDA 11.7 on an NVIDIA RTX 4090 GPU.

After training, we extracted a 2048-dimensional feature vector from the global average pooling layer. Rather than using an end-to-end classifier, we reduced these feature vectors to 512 dimensions via principal component analysis (PCA), followed by feature selection using the least absolute shrinkage and selection operator (LASSO). These steps acted as implicit regularization, further constraining model complexity. To prevent data leakage, the PCA projection was computed from the training data only and then applied to the held-out test set. To address inherent scaling differences, all extracted features subsequently underwent Z-score normalization. This systematic approach generated a standardized feature space for subsequent predictive modeling.

### 2.5 Feature selection and dimensionality reduction

This study extracted a large number of traditional radiomics and deep learning features, far exceeding the sample size, including numerous highly correlated, redundant, or irrelevant features. Direct input of all features into the model would likely induce overfitting, thereby reducing the model’s generalization ability and robustness (32–35).

Strict compartmentalization of the test data was maintained by performing all variable selection and dimensionality reduction procedures within the training set alone. The complete feature-selection procedure was performed independently for each regional feature configuration (T, TP3, TP6, and TP9). Initial filtering relied on independent t-tests and Mann-Whitney U tests to identify features with significant differences (P < 0.05) between patients achieving pCR and those who did not. To mitigate multicollinearity, Spearman’s rank correlation was employed, and redundant features with |ρ| > 0.90 were excluded. The feature selection process was finalized using the LASSO framework. This L1-regularization method automatically shrinks the coefficients of irrelevant variables to zero. Using 10-fold cross-validation to determine the optimal lambda (λ) penalty, the final model was constructed solely from features with non-zero coefficients.

### 2.6 Model construction, hyperparameter tuning, and comprehensive evaluation

#### 2.6.1 Multi-region radiomics modeling and algorithm comparison

To mitigate the risk of classifier-specific bias in selecting a candidate peritumoral margin, four machine learning algorithms—logistic regression (LR), support vector machine (SVM), random forest (RF), and extreme gradient boosting (XGBoost)—were employed for comparative evaluation. The peritumoral margin that consistently yielded the highest or near-highest cross-validated performance across all four classifiers was selected as the candidate margin for subsequent modeling, with selection performed exclusively within the training set. The held-out test set was not used at any point during margin selection and was reserved for final evaluation after the candidate margin had been fixed.

#### 2.6.2 Establishment of the clinical-radiomics and respective independent models

Independent prognostic factors, systematically identified using univariable and multivariable logistic regression, were used to construct the clinical model (CM). Concurrently, the deep learning predictive model was developed entirely based on the previously extracted deep learning-derived features. Furthermore, a combined clinical-radiomics model was built by integrating the independent clinical predictors with the selected radiomic features. To facilitate a fair performance comparison with the radiomics model under the same classifier framework, the clinical, deep learning, and combined models were all constructed utilizing the selected machine learning algorithm identified during the radiomics modeling phase. Additionally, the overarching modeling pipeline and the test set evaluation strategy remained identical across all models.

#### 2.6.3 Combined model (nomogram) and performance evaluation

We integrated the selected radiomic features, DL features, and clinical predictors to construct a combined model using LR, which was subsequently visualized via a nomogram. LR was chosen for the final integration step for three primary reasons. First, its linear structure yields high interpretability, which is essential for clinical nomograms. Second, the low-dimensional fused inputs—consisting solely of the radiomics score, DL score, and several clinical predictors—render complex nonlinear classifiers unnecessary. Finally, the continuous probability output by LR can be directly used for calibration curve and decision curve analysis (DCA), thereby enabling robust evaluation of clinical utility.

#### 2.6.4 Optimization strategies and evaluation metrics

All models followed a standardized and leakage-controlled modeling pipeline. To clarify the analysis workflow and strictly prevent information leakage, all procedures were organized into three explicitly separated stages (**Supplementary Figure 1**). First, several operations were performed on the full training set to define a fixed feature space. These operations included univariate filtering (t-test/Mann-Whitney U), Spearman-based redundancy removal, LASSO feature selection, and PCA projection for deep-learning features. Second, the Synthetic Minority Oversampling Technique (SMOTE), feature scaling, and hyperparameter grid search were performed within each cross-validation fold. SMOTE was applied only to the training partition of each fold, whereas scaling parameters estimated from that partition were applied to the corresponding held-out validation fold. Cross-validation served to select the model, hyperparameters, and the peritumoral margin. Third, for final test-set evaluation, the held-out test set was used only once, after all transformations had been fixed using the training data. We performed 5-fold stratified cross-validation on the training set, combined with grid search for hyperparameter optimization. To address class imbalance (pCR rate: 31.5%), the SMOTE algorithm was applied only within each training fold during 5-fold cross-validation, ensuring generated samples were derived solely from training data without contaminating the inner validation fold or held-out test set. Preprocessing parameters (e.g., normalization) were also tuned exclusively on the training set to strictly avoid data leakage. Feature selection and PCA were performed once on the full training set rather than within each cross-validation fold. Consequently, the cross-validation estimates used for margin, model, and hyperparameter selection may be optimistic relative to those obtained from a fully nested pipeline. However, the held-out test set was excluded from these procedures and was evaluated only after all modeling decisions were finalized; thus, its performance estimates were not directly affected by within-training-set information reuse. Model performance was quantified using AUC (compared via the DeLong test), accuracy, sensitivity, specificity, and other metrics. Threshold-dependent metrics were computed at a cutoff determined on the training set by the Youden index, which was then fixed and applied to the test set; AUC was reported as a threshold-independent metric. Calibration curves and Hosmer–Lemeshow test were applied to evaluate the consistency of predicted versus actual values. Confusion matrices and waterfall plots were generated to visualize the model’s classification performance and the distribution of individual predicted probabilities, thereby facilitating a multidimensional performance evaluation ranging from overall classification to individual risk. Decision curve analysis (DCA) was used to calculate the net benefit at different threshold probabilities and evaluate the clinical applicability of the model. This study’s workflow is shown in **Figure 3**.

**Figure 3:**
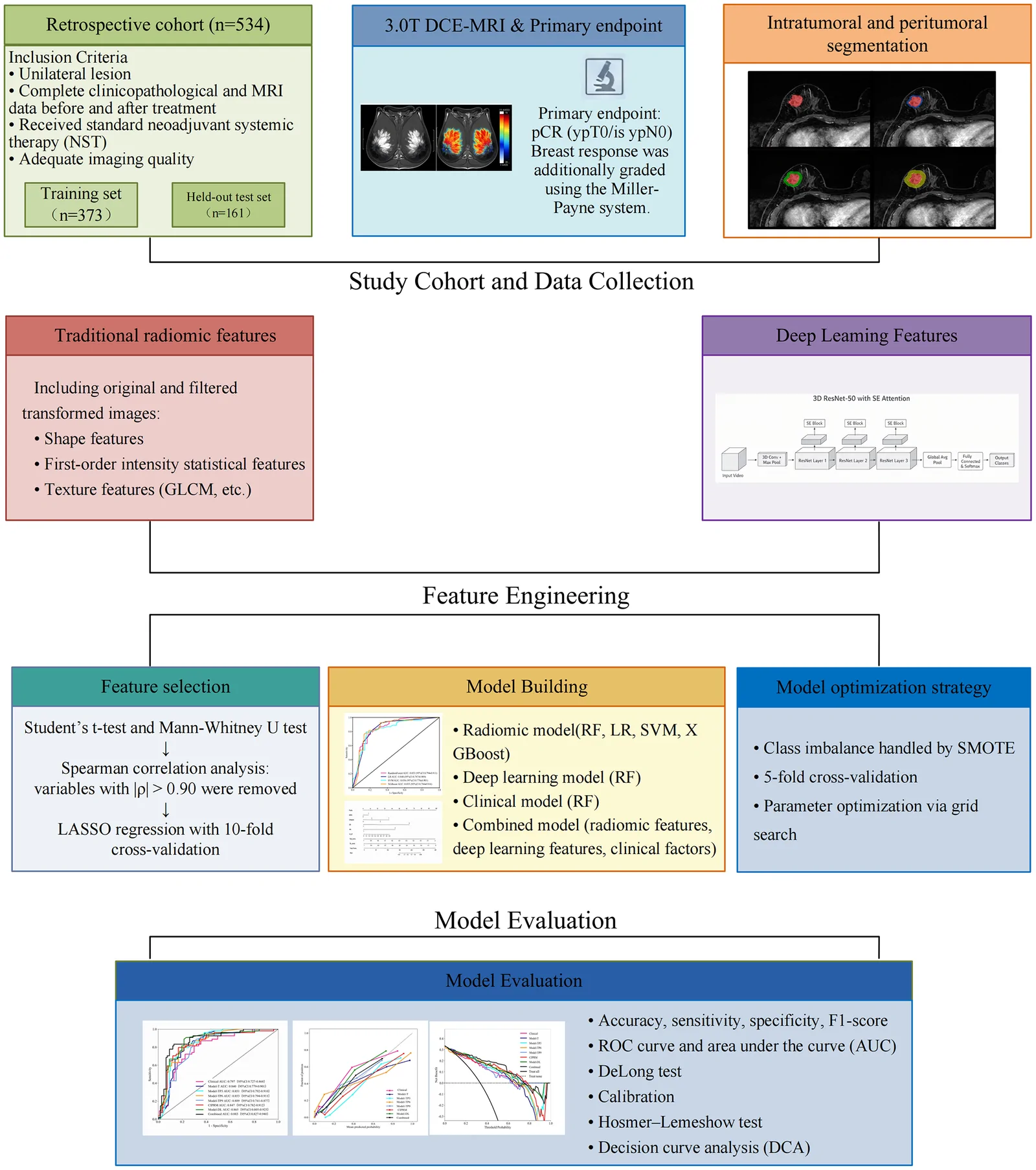
Experimental workflow, including data acquisition and preprocessing, feature extraction, feature selection, model construction, and evaluation.

### 2.7 Statistical analysis

All computational procedures and statistical analyses were performed using Python 3.7 and the statsmodels package. Statistical methods for continuous variables were selected based on distribution: independent Student’s t-tests were used for normally distributed data (presented as mean ± standard deviation), while the Mann-Whitney U test was applied for skewed data (reported as median and interquartile range). For categorical variables, differences in proportion were analyzed using the chi-square test or Fisher’s exact test, as appropriate. Univariable and multivariable logistic regression analyses were performed to identify clinical covariates with independent prognostic value. A P value < 0.05 was considered statistically significant for all comparisons.

## 3 Results

### 3.1 Analysis of clinicopathological features

#### 3.1.1 Sample distribution and intergroup balance

This study included 534 patients, with an overall pCR rate of 31.5% (168/534) and a non-pCR rate of 68.5% (366/534). Samples were randomly split into a training set (n = 373, pCR rate 30.6%) and a test set (n = 161, pCR rate 33.5%) at a 7:3 ratio. The pCR rates were comparable between the two groups, and no significant differences were observed in baseline characteristics (all P > 0.05; **Table 1**). Across molecular subtypes, the pCR rate varied substantially: HER2-enriched (68.5%, 61/89), triple-negative (34.9%, 22/63), Luminal B (26.8%, 85/317), and Luminal A (0%, 0/65). This subtype dependence of treatment response is well recognized, and the absence of pCR in Luminal A is consistent with its characteristically low chemosensitivity.

**Table 1:** Baseline characteristics.

Characteristics	Total	Training set (n = 373)			Total	Test set (n = 161)			P value^†^
		Non-pCR (n=259)	pCR (n=114)	P value^*^		Non-pCR (n=107)	pCR (n=54)	P value^*^	
Age	51.04 ± 10.09	51.28 ± 10.23	50.51 ± 9.81	0.522	50.89 ± 13.54	50.48 ± 10.29	51.72 ± 18.46	0.880	0.398
BMI (kg/m^2^)	25.14 ± 3.68	25.46 ± 3.80	24.43 ± 3.30	0.021	25.03 ± 2.99	25.44 ± 2.75	24.23 ± 3.30	0.150	0.790
Ki-67	0.40(0.20-0.60)	0.30(0.20-0.50)	0.40(0.30-0.70)	<0.001	0.40(0.20-0.60)	0.4(0.20-0.50)	0.40(0.30-0.60)	0.024	0.673
Maximum (mm)	48.97 ± 22.30	49.14 ± 22.49	48.57 ± 21.97	0.907	47.10 ± 23.05	46.81 ± 21.07	47.67 ± 26.74	0.781	0.269
Menstrual status				0.742				0.739	0.297
Menstrual cycle status	170(45.6)	120(46.3)	50(43.9)		82(50.9)	53(49.5)	29(53.7)		
Menopausal status	203(54.4)	139(53.7)	64(56.1)		79(49.0)	54(50.5)	25(46.3)		
T stage				0.445				0.246	0.282
1	19(5.1)	10(3.9)	9(7.9)		14(8.7)	6(5.6)	8(14.8)		
2	190(50.9)	134(51.7)	56(49.1)		84(52.2)	57(53.3)	27(50.0)		
3	144(38.6)	101(39.0)	43(37.7)		58(36.0)	40(37.4)	18(33.3)		
4	20(5.4)	14(5.4)	6(5.3)		5(3.1)	4(3.7)	1(1.9)		
N stage				0.193				0.729	0.861
0	60(16.1)	38(14.7)	22(19.3)		24(14.9)	15(14.0)	9(16.7)		
1	65(17.4)	52(20.1)	13(11.4)		33(20.5)	24(22.4)	9(16.7)		
2	197(52.8)	135(52.1)	62(54.4)		82(50.9)	55(51.4)	27(50.0)		
3	51(13.7)	34(13.1)	17(14.9)		22(13.7)	13(12.2)	9(16.7)		
ER status				<0.001				<0.001	0.707
Positive	258(69.2)	205(79.1)	53(46.5)		108(67.1)	86(80.4)	22(40.7)		
Negative	115(30.8)	54(20.9)	61(53.5)		53(32.9)	21(19.6)	32(59.3)		
PR status				<0.001				<0.001	0.774
Positive	229(61.4)	187(72.2)	42(36.8)		96(59.6)	75(70.1)	21(38.9)		
Negative	144(38.6)	72(27.8)	72(63.2)		65(40.4)	32(29.9)	33(61.1)		
HER2 status				<0.001				<0.001	0.636
Positive	150(40.2)	70(27.0)	80(70.2)		69(42.9)	28(26.2)	41(75.9)		
Negative	223(59.8)	189(73.0)	34(29.8)		92(57.1)	79(73.8)	13(24.1)		
Histological grade				0.002				0.169	0.293
I	2(0.5)	2(0.8)	/		/	/	/		
II	299(80.2)	219(84.5)	80(70.2)		133(82.6)	89(83.2)	44(81.5)		
III	67(18.0)	37(14.3)	30(26.3)		23(14.3)	13(12.1)	10(18.5)		
II-III	5(1.3)	1(0.4)	4(3.5)		5(3.1)	5(4.7)	/		
Lymphatic metastasis				0.333				0.833	0.831
Metastasis	313(83.9)	221(85.3)	92(80.7)		137(85.1)	92(86.0)	45(83.3)		
No metastasis	60(16.1)	38(14.7)	22(19.3)		24(14.9)	15(14.0)	9(16.7)		

*P-values for comparisons between the pCR and non-pCR groups within each cohort.

^†^P-values for comparisons between the training and test sets to assess baseline balance between the two cohorts.

#### 3.1.2 Screening for independent predictive factors

In univariate logistic regression, ER, PR, HER2, Ki-67, BMI, and histological grade were significantly associated with pCR status, whereas age, T stage, N stage, maximum tumor diameter, menstrual status, and lymph node status showed no significant association. In multivariable logistic regression, BMI (OR 1.008, P = 0.545) and histological grade (OR 1.066, P = 0.202) did not retain significance after adjustment and were consequently excluded. Therefore, ER, PR, HER2, and Ki-67 were identified as the four independent predictors of pCR (P < 0.05). As shown in **Figure 4**, these four independent predictors were ultimately used to construct the clinical and combined models.

**Figure 4:**
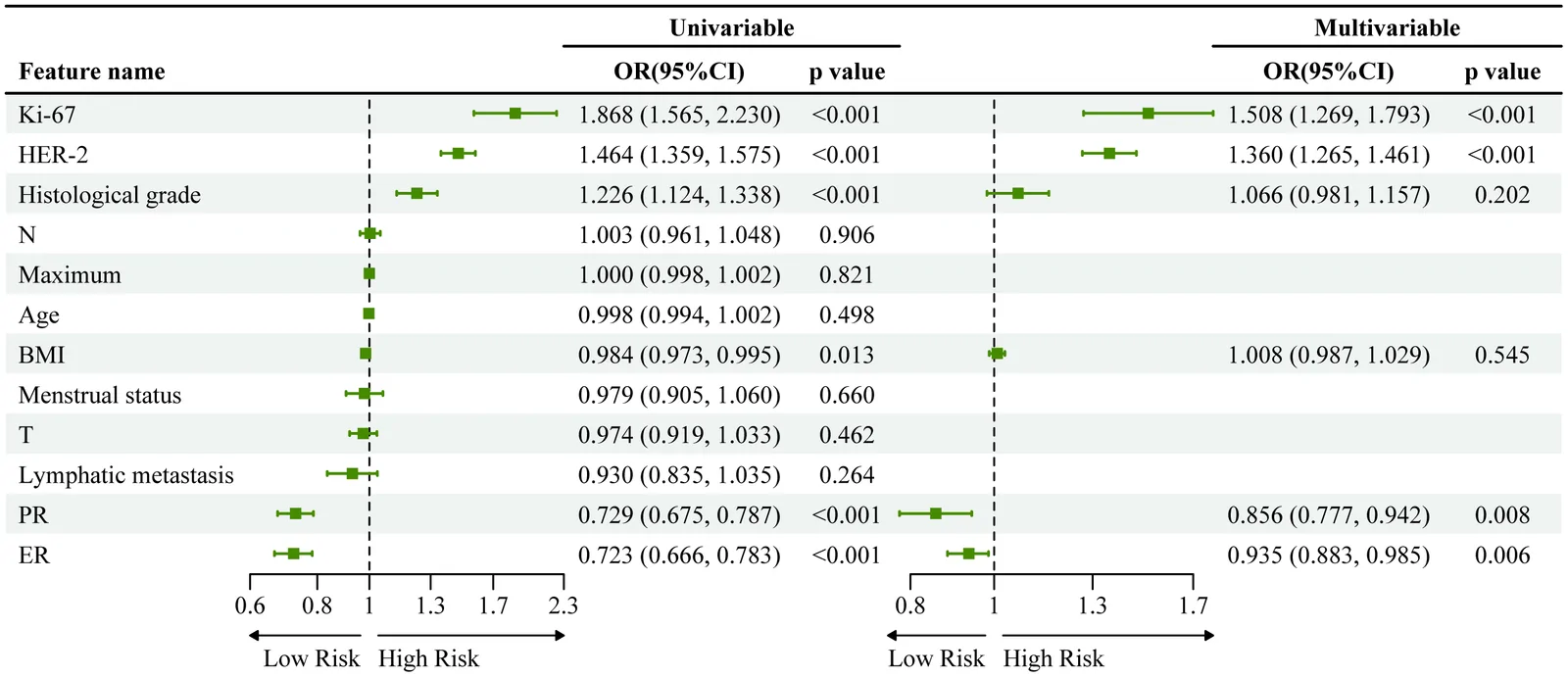
Forest plot of independent factors for predicting pCR status based on clinical predictors of breast cancer in the training set.

### 3.2 Feature selection and model construction

#### 3.2.1 Radiomics feature selection

A total of 1,197 candidate radiomic features were obtained for each evaluated region, including the intratumoral region and the intratumoral plus 3-mm, 6-mm, and 9-mm peritumoral regions. These features comprised first-order features (n=234), GLCM (n=286), GLDM (n=182), GLRLM (n=208), GLSZM (n=208), NGTDM (n=65), and shape features (n=14).

The feature selection procedure was performed as follows: initial screening was conducted using the t-test or Mann-Whitney U test, followed by Spearman correlation analysis to remove redundant features, which markedly reduced the dimensionality and collinearity of the feature space. For key feature selection, LASSO regression was applied (**Figure 5**), and 13–14 key features were ultimately identified for each model (**Figure 6**; **Supplementary Table 1**).

**Figure 5:**
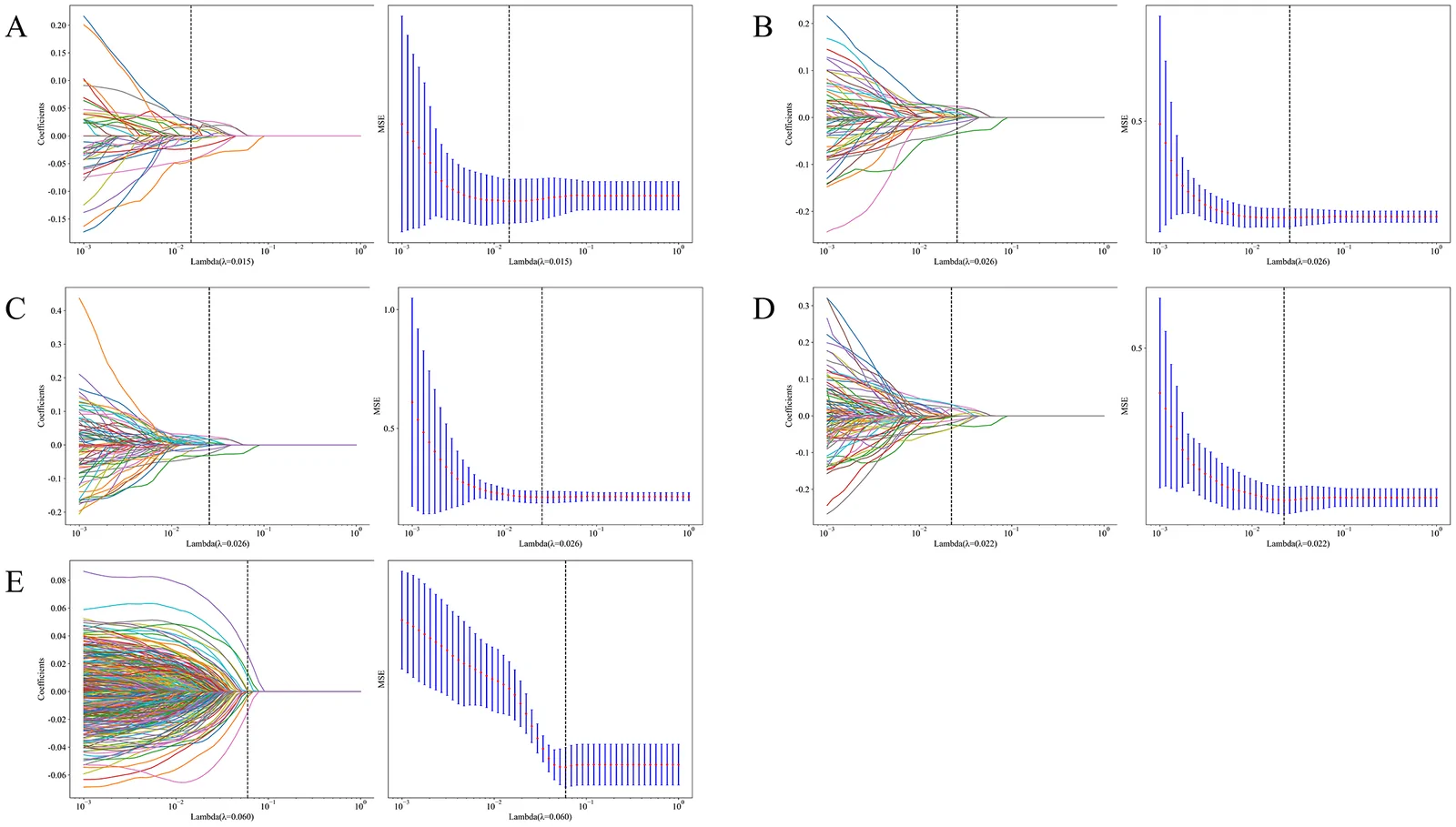
LASSO coefficient trajectory plot of radiomic and deep learning features and cross-validation mean squared error (MSE) curve of LASSO regression under 10-fold cross-validation. Each curve represents the trajectory of the regression coefficient for a single feature as a function of log(λ). The vertical dashed line indicates the optimal λ value determined by the minimum criterion, the red dashed line marks the MSE corresponding to the optimal λ value, and the blue vertical bars represent the confidence intervals for the MSE estimates. **(A)** Intratumoral features; **(B)** Intratumoral plus 3-mm peritumoral features; **(C)** Intratumoral plus 6-mm peritumoral features; **(D)** Intratumoral plus 9-mm peritumoral features; **(E)** Deep learning features.

**Figure 6:**
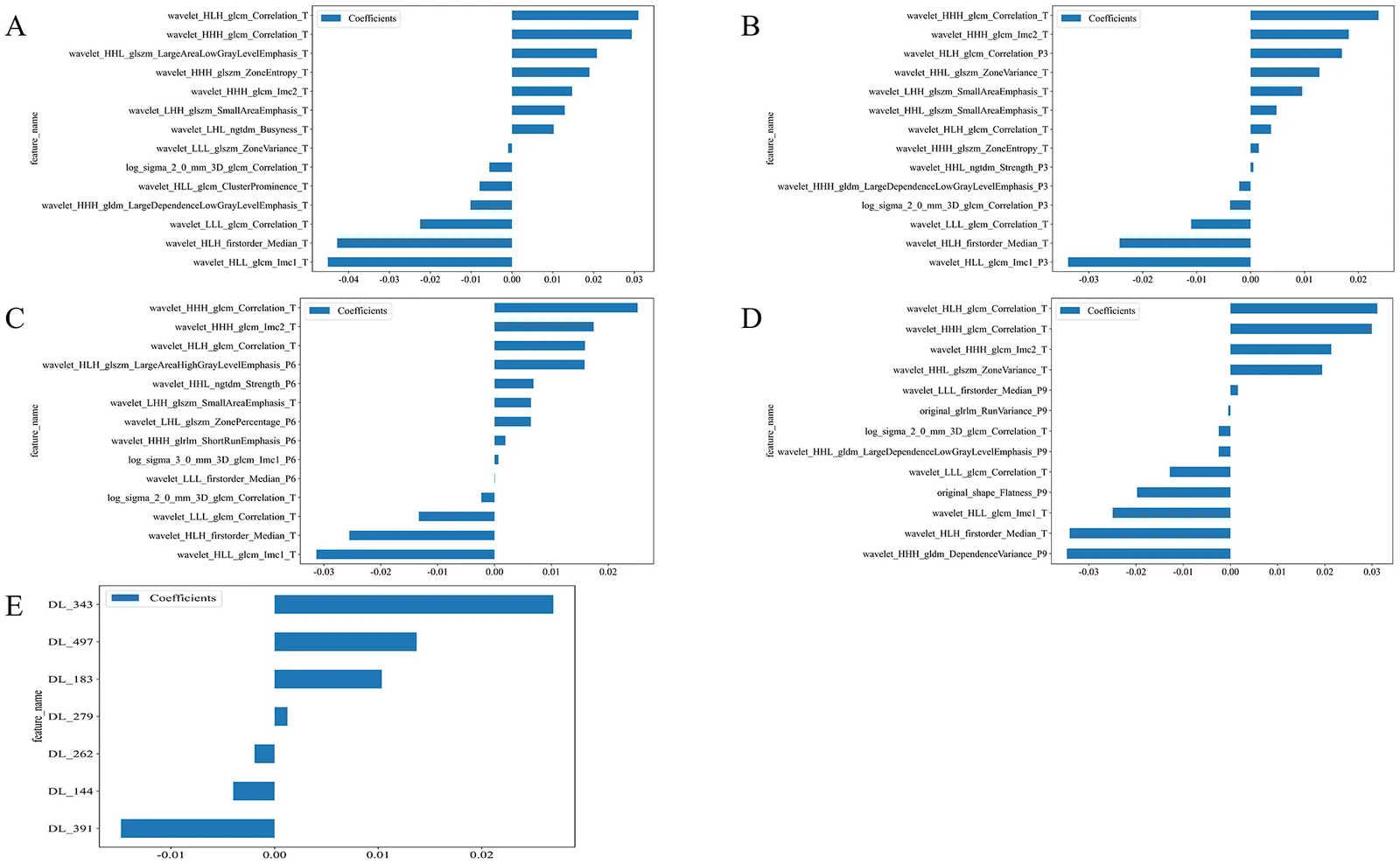
Radiomic and deep learning features with non-zero coefficients retained by LASSO at the selected λ. The bar plot shows the (standardized) regression coefficients, with positive and negative values indicating the direction of association, respectively. **(A)** Intratumoral features; **(B)** Intratumoral plus 3-mm peritumoral features; **(C)** Intratumoral plus 6-mm peritumoral features; **(D)** Intratumoral plus 9-mm peritumoral features; **(E)** Deep learning features.

#### 3.2.2 Multi-algorithm comparison and training-set selection of the candidate peritumoral margin

This study employed four machine learning algorithms to evaluate the predictive performance of models across different regions (**Figure 7**, **Table 2**; **Supplementary Figure 2**; **Supplementary Table 2**). To prevent information leakage from the test set, the candidate peritumoral margin was selected exclusively within the training set. In the training set, Model-TP6 yielded the highest AUCs across all classifiers (RF: 0.880, 95% CI: 0.846-0.915; LR: 0.893, 95% CI: 0.859-0.927; SVM: 0.912, 95% CI: 0.882-0.942; XGBoost: 0.901, 95% CI: 0.867-0.935), showing numerically higher AUCs than Model-TP3 and Model-TP9 (the latter showing a decline to 0.834-0.849). Based on these training-set results, the intratumoral plus 6-mm peritumoral margin was selected as the candidate spatial scope for subsequent modeling prior to test-set evaluation. Subsequently, the training-set-selected Model-TP6 was evaluated in the held-out test set to provide an estimate of within-center performance. Using the RF algorithm, Model-TP6 achieved a test-set AUC of 0.853 (95% CI: 0.794-0.911), compared with 0.840 for Model-T and 0.851 for Model-TP3. Notably, Model-TP6 yielded the numerically highest or near-highest AUC across all four classifiers, suggesting that the intratumoral plus 6-mm peritumoral margin showed the most favorable numerical performance among the evaluated regions. However, the pairwise DeLong comparison between Model-TP6 and Model-T did not reach statistical significance (**Figure 8**, DeLong P = 0.358; Section 3.3.1). Therefore, the observed difference should be interpreted as a numerical trend rather than a statistically confirmed advantage.

**Figure 7:**
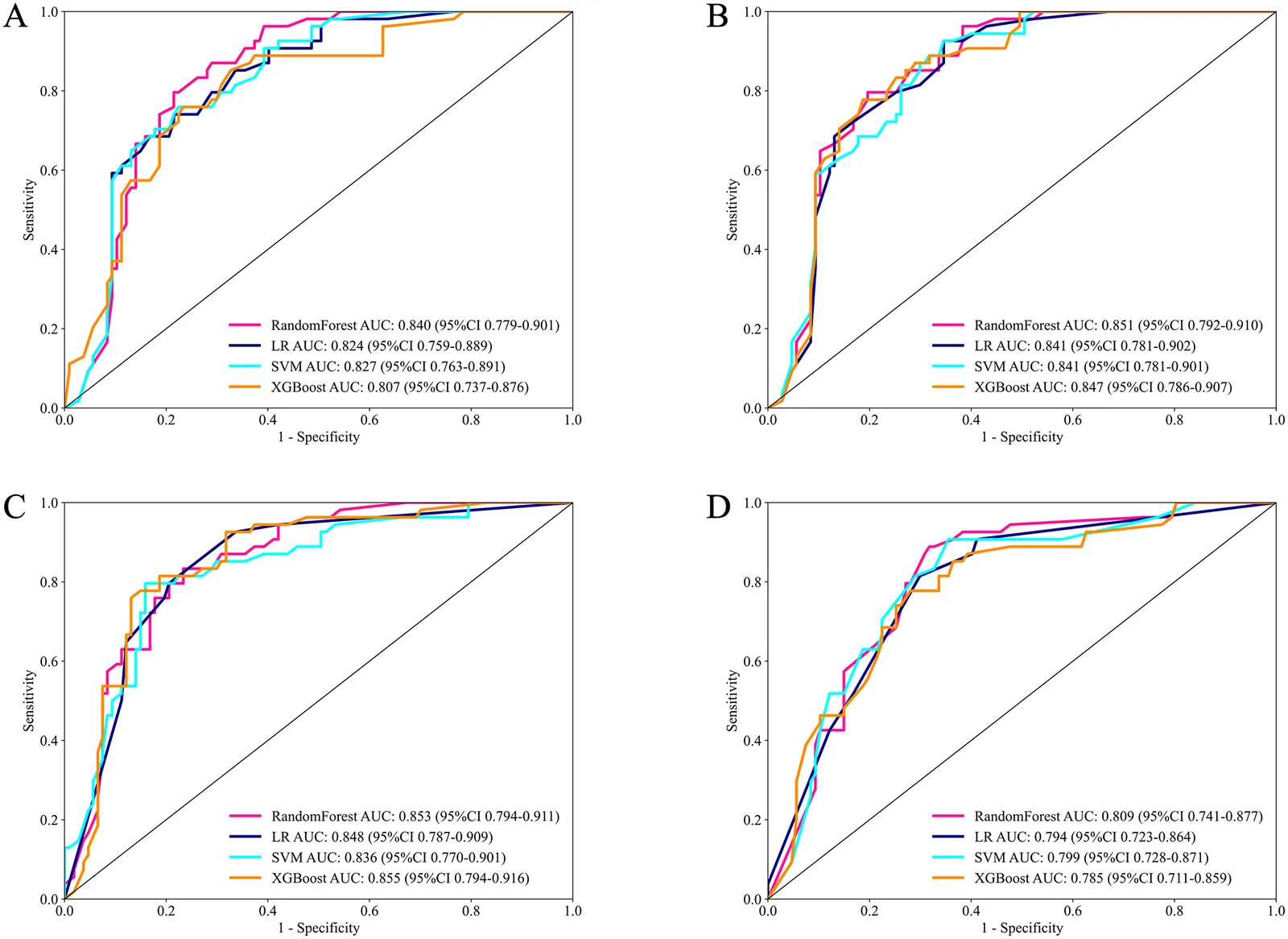
Comparison of ROC curves of machine learning models based on feature sets from different regions in the test set. **(A)** Model-T; **(B)** Model-TP3; **(C)** Model-TP6; **(D)** Model-TP9.

**Table 2:** Comparison of predictive performance of machine learning models based on feature sets from different regions in the test set.

Regional model	Classifier	AUC	95% CI	Acc	Sen	Spe	F1
Model-T	RF	0.840	0.779-0.901	0.789	0.796	0.785	0.717
	LR	0.824	0.759-0.889	0.770	0.741	0.785	0.684
	SVM	0.827	0.763-0.891	0.770	0.759	0.776	0.689
	XGBoost	0.807	0.737-0.876	0.764	0.759	0.766	0.683
Model-TP3	RF	0.851	0.792-0.910	0.801	0.796	0.804	0.729
	LR	0.841	0.781-0.902	0.745	0.926	0.654	0.709
	SVM	0.841	0.781-0.901	0.745	0.926	0.654	0.709
	XGBoost	0.847	0.786-0.907	0.801	0.778	0.813	0.724
Model-TP6	RF	0.853	0.794-0.911	0.789	0.833	0.766	0.726
	LR	0.848	0.787-0.909	0.795	0.796	0.794	0.723
	SVM	0.836	0.770-0.901	0.826	0.796	0.841	0.754
	XGBoost	0.855	0.794-0.916	0.832	0.759	0.869	0.752
Model-TP9	RF	0.809	0.741-0.877	0.752	0.889	0.682	0.706
	LR	0.794	0.723-0.864	0.739	0.815	0.701	0.677
	SVM	0.799	0.728-0.871	0.733	0.907	0.645	0.695
	XGBoost	0.785	0.711-0.859	0.739	0.778	0.720	0.667

**Figure 8:**
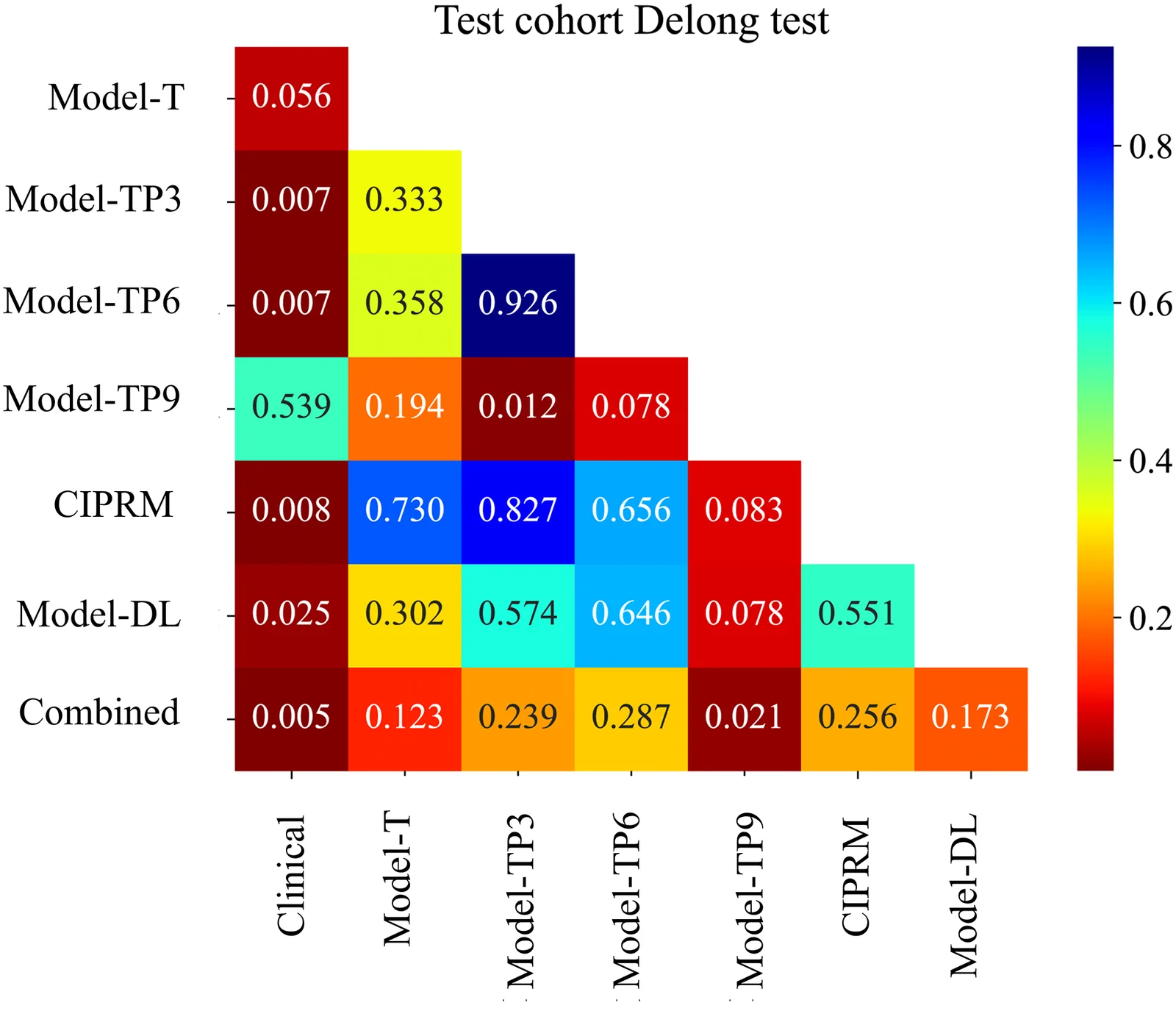
DeLong test for eight models in the test set.

To enable fair head-to-head comparisons among the subsequently developed clinical, deep learning, and clinical-radiomics models, a unified classifier was required. RF was adopted for this purpose given its balanced performance across all regional feature sets (**Supplementary Figure 2**; **Supplementary Table 2**). Of note, this choice pertains only to the individual model comparison stage; the final combined nomogram was constructed using logistic regression to ensure clinical interpretability, as detailed in Section 2.6.3.

#### 3.2.3 Selection and construction of deep learning features and models

Based on 512 DL features extracted from the training-set-selected TP6 region and using the same screening procedure as traditional radiomics methods, seven core DL features were retained (detailed coefficients are listed in **Supplementary Table 1**; the selection trajectory and MSE curves are presented in **Figure 5E**; **Figure 6E**). These seven selected features formed the input feature set for the DL model.

The DL model constructed using the RF algorithm showed favorable predictive performance. In the test set, the model achieved an AUC of 0.865 (95% CI: 0.805–0.925), and provided incremental value over the traditional radiomics model (Model-TP6) in accuracy (0.820), sensitivity (0.889), and specificity (0.785) (**Figure 9**; **Table 3**). Calibration curves demonstrated that the DL model achieved a better fit, with predicted probabilities highly consistent with actual observations (**Figure 10**). DCA further showed that the DL model conferred a higher net benefit than Model-TP6 in the test set, particularly within the lower-to-intermediate threshold range, while retaining favorable clinical predictive value (**Figure 11**).

**Figure 9:**
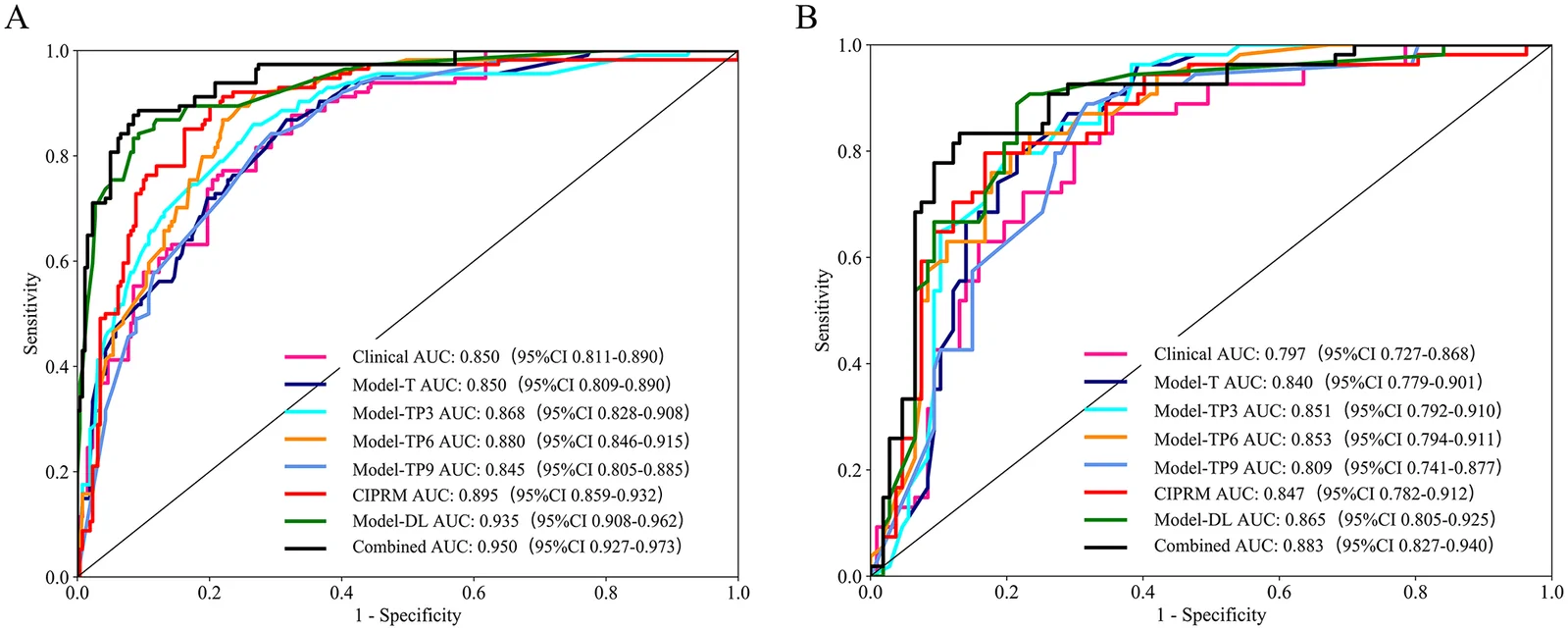
ROC curves of all models in the training and test sets. **(A)** Training set; **(B)** Test set.

**Table 3:** Performance summary of all models in the training and test sets.

Model	Cohort	AUC	95% CI	Acc	Sen	Spe	F1
CM	Training set	0.850	0.811 - 0.890	0.735	0.868	0.676	0.667
	Test set	0.797	0.727 - 0.868	0.733	0.796	0.701	0.667
Model-TP6	Training set	0.880	0.846 - 0.915	0.788	0.921	0.730	0.727
	Test set	0.853	0.794 - 0.911	0.789	0.833	0.766	0.726
Model-DL	Training set	0.935	0.908 - 0.962	0.887	0.842	0.907	0.821
	Test set	0.865	0.805 - 0.925	0.820	0.889	0.785	0.768
CIPRM	Training set	0.895	0.859 - 0.932	0.826	0.886	0.799	0.757
	Test set	0.847	0.782 - 0.912	0.814	0.778	0.832	0.737
Combined model	Training set	0.950	0.927 - 0.973	0.898	0.877	0.907	0.840
	Test set	0.883	0.827 - 0.940	0.851	0.815	0.869	0.786

**Figure 10:**
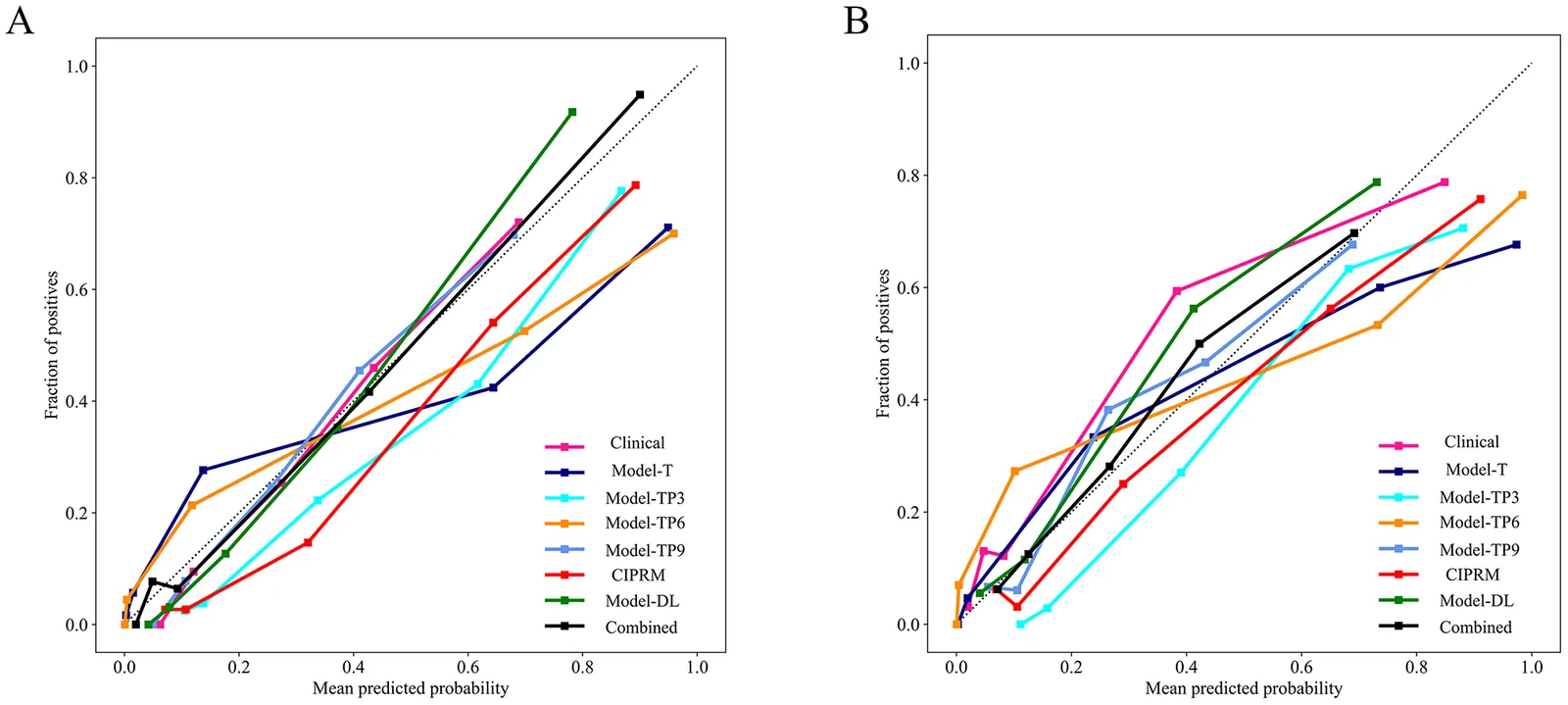
Calibration curves of all models in the **(A)** training set and **(B)** test set. The x-axis represents the predicted probability, and the y-axis represents the observed frequency of outcome occurrence. The 45° diagonal line indicates ideal calibration; the closer the calibration curve is to the diagonal line, the better the model calibration.

**Figure 11:**
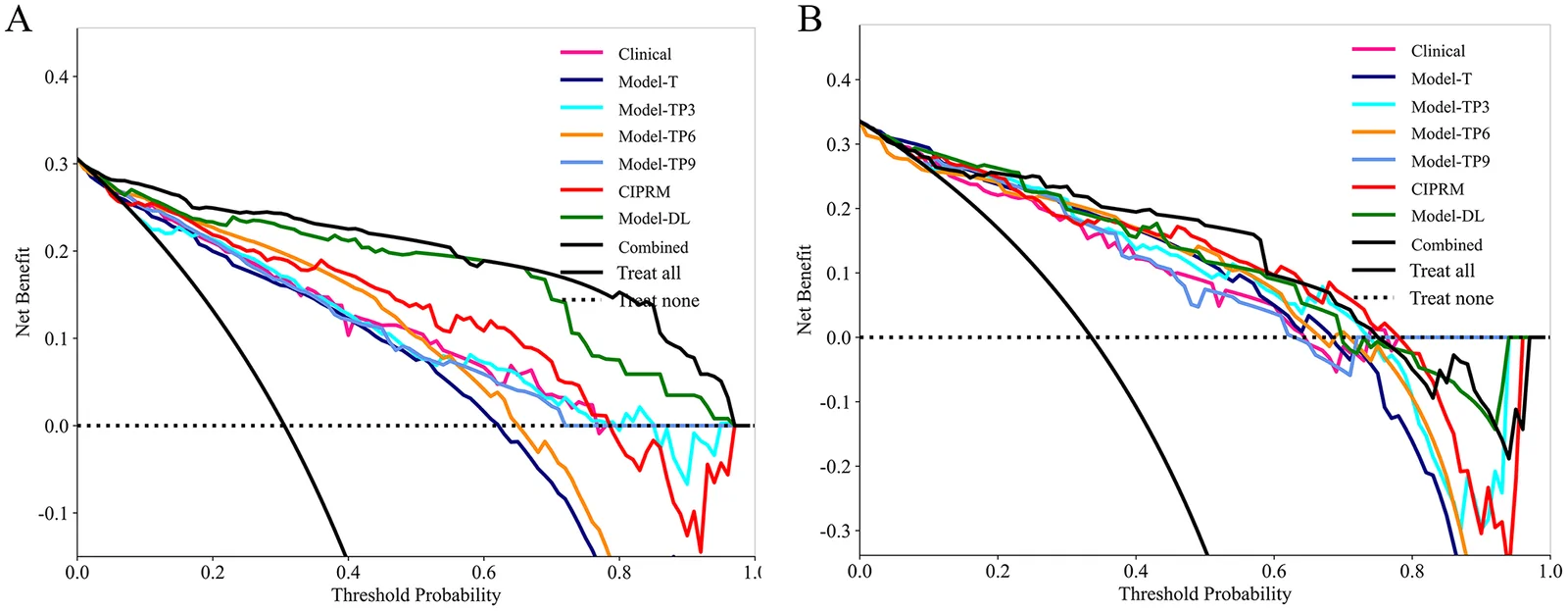
Decision curves comparing the net benefit of the models across different threshold probability ranges in the **(A)** training set and **(B)** test set. The “Treat-all” (treating all patients) and “Treat-none” (treating no patients) strategies were presented as reference lines.

#### 3.2.4 Construction of the clinical model

The clinical model was constructed using the RF algorithm based on four independent clinical predictors selected by multivariate logistic regression: ER, PR, HER2, and Ki-67 expression. In the test set, the AUC was 0.797 (95% CI: 0.727–0.868), confirming its fundamental predictive value (**Figure 4**, **Table 3**).

#### 3.2.5 Construction of the clinical and intra-peritumoral radiomics model

To assess the added value of combining clinical and radiomic information, this study integrated the radiomics features of Model-TP6 with the four aforementioned clinical predictors using an RF algorithm, establishing a Clinical and Intra-Peritumoral Radiomics Model (CIPRM). When evaluated in the test set, the CIPRM achieved an AUC of 0.847 (95% CI: 0.782–0.912), with corresponding accuracy, sensitivity, and specificity of 0.814, 0.778, and 0.832. ROC curve analysis (**Figure 9**) showed that this discrimination was numerically higher than that of the clinical-only model (0.797) and comparable to the radiomics-only Model-TP6 (0.853), with overlapping confidence intervals. The absence of a further numerical gain over Model-TP6 likely reflects partial redundancy between the clinical receptor markers and the radiomic phenotype, together with the limited test-set size (n = 161), rather than a loss of predictive information.

#### 3.2.6 Construction of the combined model

In this study, a multimodal fusion model was developed by integrating clinical factors, Model-TP6 radiomic features, and deep learning signatures. As detailed in the Methods section, the integrated model was constructed using LR. To facilitate individualized risk visualization, we further developed a visual nomogram (**Figure 12**). The nomogram presents the contribution weights of each factor for pCR prediction in the following order: radiomics score ≈ deep learning score > ER > PR > Ki-67 > HER2.

**Figure 12:**
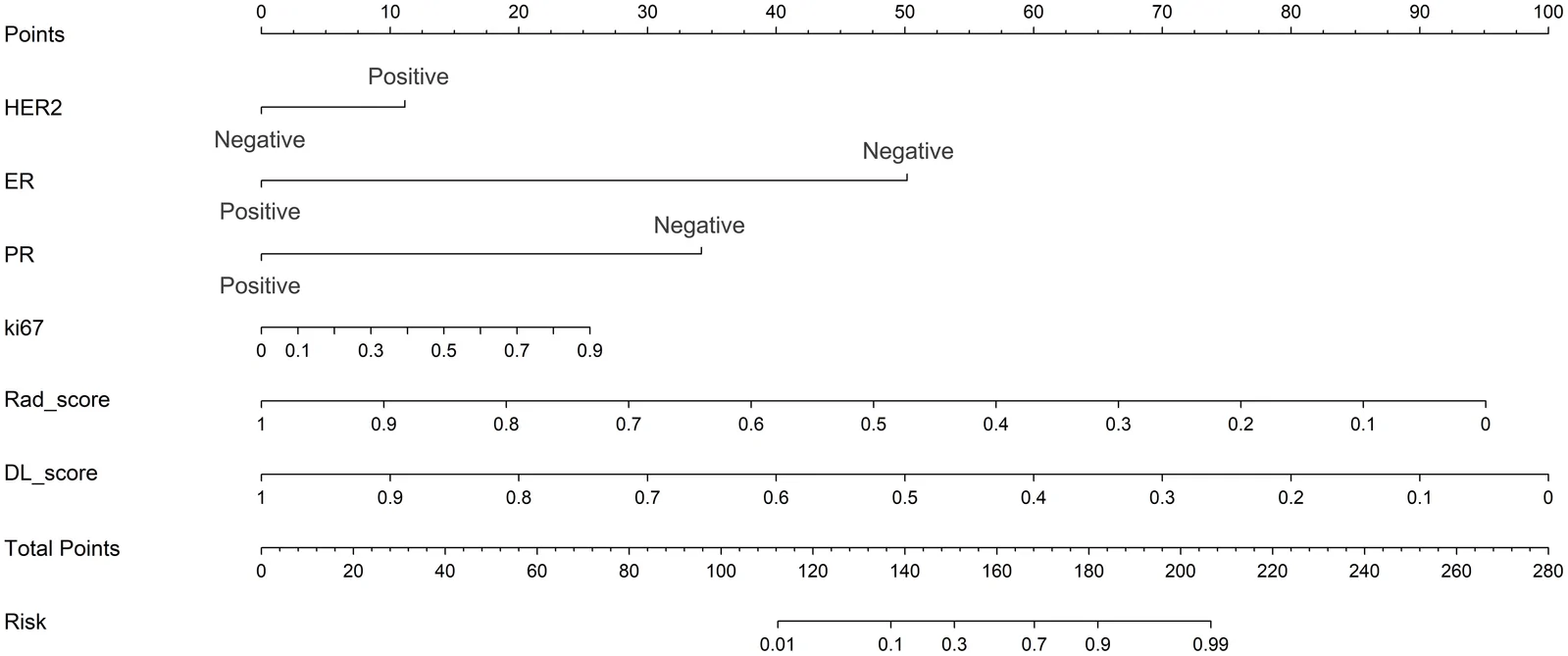
Nomogram of the combined model.

ROC curve analysis (**Figure 9**, **Table 3**) indicated that the combined model achieved the numerically highest AUC among all models. It yielded an AUC of 0.950 (95% CI: 0.927–0.973) in the training set and an AUC of 0.883 (95% CI: 0.827–0.940) in the held-out internal test set. In the test set, the model showed favorable discriminative performance, with an accuracy, sensitivity, and specificity of 0.851, 0.815, and 0.869, respectively. As all evaluation was performed by internal testing on a single-center, single-scanner cohort, the reported performance may not generalize to other settings.

### 3.3 Model performance evaluation

#### 3.3.1 DeLong test

Although the limited test-set size (n = 161) may have reduced the statistical power of the pairwise DeLong comparisons, Model-TP6 yielded higher AUCs than Model-T across all four classifiers. For the RF classifier, the AUCs were 0.853 and 0.840, respectively; however, this difference was not statistically significant (DeLong P = 0.358) and should therefore be interpreted as a consistent numerical trend favoring the inclusion of peritumoral features rather than a confirmed advantage. Furthermore, the fully integrated nomogram achieved the numerically highest test-set AUC (0.883, 95% CI: 0.827-0.940). The combined model significantly outperformed the clinical model (P = 0.005) and Model-TP9 (P = 0.021), whereas differences from the remaining models did not reach statistical significance. Despite these mixed results, the nomogram demonstrated a favorable combination of discrimination, calibration, and decision-curve net benefit. This supports its potential as an exploratory predictive tool, which warrants external validation.

#### 3.3.2 Calibration curves and the Hosmer–Lemeshow test

As visually represented in the calibration plots and further assessed using Hosmer-Lemeshow (H-L) tests (**Figure 10**, **Table 4**), the combined model achieved favorable goodness-of-fit. Its predicted probabilities showed reasonable agreement with the 45-degree reference line across both the training (P = 0.274) and internal test (P = 0.115) cohorts. Conversely, Model-TP3 showed evidence of poor calibration in both cohorts (P < 0.001). Beyond the Hosmer-Lemeshow test, we evaluated the calibration slope, intercept, and Brier score (**Supplementary Table 3**). In the training cohort, the model showed good calibration (slope 1.220, intercept 0.114, Brier score 0.073). In the held-out internal test cohort, the calibration slope was 0.818 and the intercept was 0.328, indicating mild calibration drift with a slight tendency toward more extreme and modestly underestimated probabilities, while the Brier score remained low (0.133). These metrics indicate imperfect probabilistic reliability within this single-center cohort, reinforcing the need for recalibration and external validation.

**Table 4:** Hosmer–Lemeshow test results for all models.

Cohort	Clinical Model	Model-T	Model-TP3	Model-TP6	Model-TP9	CIPRM	DL model	Combined model
Training	0.014	0.056	<0.001	0.077	0.954	0.099	0.081	0.274
Test	0.701	0.063	<0.001	0.069	0.180	0.091	0.175	0.115

#### 3.3.3 Confusion matrix and waterfall plot

The confusion matrix (**Figure 13A**) was further employed to evaluate the classification performance. Among the 161 samples, the model correctly identified 93 non-pCR patients (TN) and 44 pCR patients (TP), with 14 false positives (FP) and 10 false negatives (FN). This yielded an accuracy of 85.1%, a sensitivity of 81.5%, and a specificity of 86.9%. The corresponding 95% confidence intervals for accuracy, sensitivity, specificity, positive predictive value (PPV), and negative predictive value (NPV) of the combined model in the test set are provided in **Supplementary Table 4** (e.g., accuracy 0.851, 95% CI: 0.788–0.898; PPV 0.759, 95% CI: 0.635–0.850; NPV 0.903, 95% CI: 0.830–0.946). These results indicate relatively balanced predictive performance: high specificity supports reliable identification of non-pCR patients, while maintaining adequate sensitivity for pCR cases.

**Figure 13:**
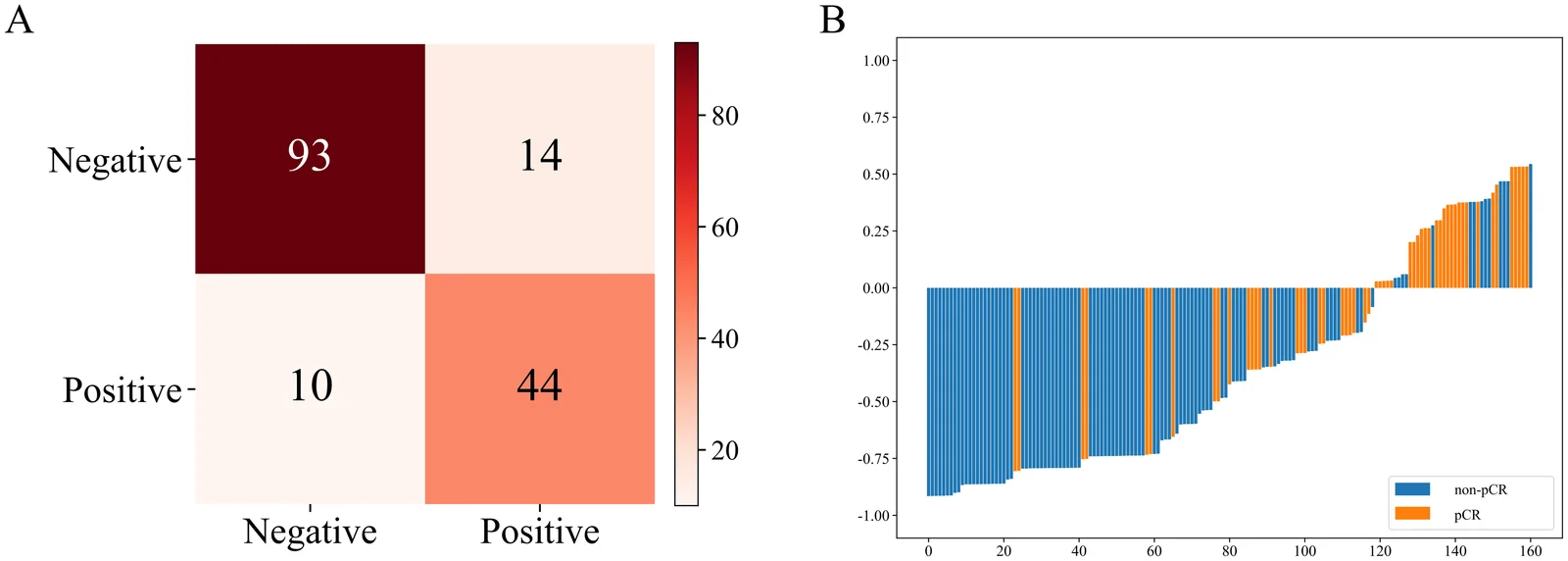
Classification performance of the combined model in the test set. **(A)** Confusion matrix of the combined model for pCR prediction. The matrix shows the numbers of true positives, true negatives, false positives, and false negatives in the test set; **(B)** Waterfall plot of model-predicted logit scores for patients, ranked from lowest to highest. Different colors represent pCR and non-pCR patients, illustrating the distribution of logit scores across groups with different pathological responses.

Additionally, to further investigate predictive performance at the individual level, we constructed a waterfall plot of prediction scores (**Figure 13B**). Overall, patients with pathological complete response (pCR) tended to have higher prediction scores, whereas non-pCR patients generally exhibited lower scores, showing high consistency with the gold standard. The 24 misclassified cases (predominantly false positives) had low scores close to the zero threshold, suggesting that these cases were in the region of low prediction certainty rather than being definitively predicted. By clearly visualizing these discrepant cases, the plot illustrates individual prediction deviations and areas of model uncertainty.

#### 3.3.4 Decision curve analysis

DCA (**Figure 11**) showed that, across much of the clinically relevant threshold range of approximately 0.2–0.6, the combined model achieved a higher net benefit than the treat-all and treat-none strategies as well as the other single models, in both the training and internal test sets. This suggests that the model has potential utility for effective risk stratification as an exploratory research tool, pending future external validation.

## 4 Discussion

### 4.1 Key findings

pCR serves as a critical surrogate endpoint for treatment efficacy and long-term prognosis in NST for breast cancer, particularly in HER2-enriched and triple-negative subtypes (2, 4). Accurate pretreatment prediction of pCR could potentially inform future clinical decision-making within a defined decision context. Within the evaluated threshold range of approximately 0.2–0.6, DCA showed that the combined model provided a higher estimated net benefit than the treat-all and treat-none strategies across much of this range (**Figure 11**). If prospectively validated in multicenter cohorts, the model may help identify candidates for treatment de-escalation or closer monitoring and may inform treatment adjustment and individualized surgical planning. These potential applications were not evaluated in the present study; therefore, the nomogram should currently be considered an exploratory research tool rather than a basis for clinical decisions. The heterogeneity of breast cancer leads to substantial interindividual variability in NST response. Traditional imaging assessment often emphasizes morphological changes and may not fully capture treatment-related heterogeneity, potentially delaying response assessment. Accordingly, noninvasive pretreatment prediction of pCR remains a clinically relevant research objective.

The combined model of radiomics, deep learning, and clinical predictors developed in this study exhibited strong performance, with AUCs of 0.950 and 0.883 in the training and test sets, respectively, with numerically higher AUCs than the individual models. The present study also found that the 6-mm peritumoral region showed the most favorable numerical performance, suggesting a potential contribution of the tumor microenvironment in reflecting tumor invasiveness and treatment response. Model comparison demonstrated that the deep learning model (AUC = 0.865) showed a numerically higher AUC than the traditional radiomic model (AUC = 0.853), suggesting a potential advantage of deep learning in extracting high-level complex features.

Among clinical baseline characteristics, ER, PR, HER2 status, and Ki-67 were identified as independent predictors of pCR (**Figure 4**). The present study confirmed that higher baseline Ki-67 levels were associated with a higher pCR rate. As a key marker of cell proliferation activity (36), higher Ki-67 expression is associated with aggressive tumor biology. However, this highly proliferative state may also be associated with greater sensitivity to chemotherapy. This finding is highly consistent with a recent study by Toli et al. (37). Hormone receptor-positive tumors exhibit low proliferative activity and favorable prognosis, but show a poor response to neoadjuvant therapy, with a relatively low pCR rate (38–40). In contrast, although HER2-enriched breast cancer is more aggressive (41), it achieves a significantly higher pCR rate following targeted therapy such as trastuzumab (42, 43), demonstrating substantial potential for clinical benefit. Although molecular subtype is biologically associated with pCR, it is derived from receptor and proliferation markers; to avoid collinearity, it was not retained as a separate predictor in the final model, and the biological differences it reflects are largely captured by ER, PR, HER2, and Ki-67. These biological differences influence drug sensitivity and contribute to the heterogeneity of treatment response.

### 4.2 Interpretation and comparative analysis of key findings

#### 4.2.1 Evaluation of candidate peritumoral margins for pCR prediction

The peritumoral region carries substantial biological significance in tumor occurrence, progression, and treatment response. It is not a passive “blank region,” but a biologically active area abundant in information related to immune response, angiogenesis, and matrix remodeling. Previous studies, such as those by Braman et al. (20, 21), have demonstrated that peritumoral radiomic features are associated with tumor-infiltrating lymphocyte density and can predict treatment response in breast cancer. Li et al. (19) further noted that an intratumoral–peritumoral combined model yields a higher AUC than a single-region model. Nevertheless, no consensus has been reached regarding the optimal peritumoral width.

The 6-mm peritumoral margin showed the most favorable numerical performance among the evaluated margins for predicting pCR. Across all four classifiers, Model-TP6 yielded a higher AUC than Model-T (e.g., RF: 0.853 vs. 0.840; XGBoost: 0.855 vs. 0.807), although the between-model differences did not reach statistical significance in DeLong tests (e.g., Model-TP6 vs. Model-T, P = 0.358). This consistent numerical pattern suggests that peritumoral features may provide additional predictive information. Biologically, the 6-mm margin may capture the most informative region during microenvironmental remodeling—including peritumoral edema, inflammatory activation, and early angiogenic changes (44, 45)—while avoiding signal dilution from unrelated tissues at wider margins. This interpretation is consistent with the numerical performance decline observed for Model-TP9. We therefore propose that a potentially informative margin may lie within a range (e.g., 3–6 mm); finer-grained exploration (e.g., 4, 5, 7, 8 mm) in independent cohorts is warranted. Notably, the optimal peritumoral width is likely task-dependent, as Zhou et al. (46) reported 4 mm as optimal for HER2 status prediction.

#### 4.2.2 Feature correlation and classifier robustness

As shown in **Table 2** and **Figure 8**, although Model-TP6 showed favorable numerical performance, its difference from Model-TP3 was not statistically significant. These two models shared several texture features derived from the intratumoral region, such as wavelet_HHH_glcm_Correlation_T (**Supplementary Table 1**). To verify model stability, this study employed multiple machine learning algorithms (**Table 2**). The results demonstrated that Model-TP6 performed favorably overall in terms of AUC, accuracy, specificity, and F1 score across different classifiers. This indicates that the compatibility between feature combinations and classifiers is critical. Future studies may further explore ensemble learning to improve model stability (47, 48).

#### 4.2.3 Performance and rationale of multimodal fusion

CIPRM achieved an AUC of 0.847 in the test set, numerically higher than the clinical-only model and comparable to the radiomics-only model. This supports the rationale for integrating clinical predictors, intratumoral heterogeneity, and the peritumoral microenvironment into modeling: clinical predictors provide systemic background, intratumoral features characterize the tumor itself, and peritumoral features reflect microenvironmental aggressiveness. These components may provide complementary information; however, CIPRM did not demonstrate additional discrimination over Model-TP6 in the present test set.

#### 4.2.4 Comparison and integration of traditional radiomics and deep learning

This study found that the DL model showed numerically higher performance than traditional radiomics across several metrics, calibration curves, and net benefit analyses (**Table 3**, **Figures 9**–**11**). This numerical pattern may reflect DL’s powerful multilayer architecture, which automatically extracts abstract, nonlinear features and compensates for the limitations of handcrafted features in capturing complex tumor heterogeneity (27). In line with these results, Peng et al. (49) reported that DL models achieve higher AUCs for pCR prediction. However, the inherent “black-box” nature of DL limits its clinical interpretability (27, 28).

To address this interpretability limitation, we constructed a fully integrated model combining clinical variables, radiomics, and DL features. This model achieved the numerically highest test-set AUC (0.883, 95% CI: 0.827–0.940). Although it significantly outperformed the clinical model (P = 0.005) and Model-TP9 (P = 0.021), its pairwise comparisons with CIPRM (P = 0.256) and the remaining models did not reach statistical significance. Beyond discrimination, it was the only model to simultaneously demonstrate favorable calibration in both cohorts (H-L P = 0.274 and 0.115), higher DCA net benefit across much of the clinically relevant threshold range, and a balanced sensitivity–specificity trade-off. This convergence of complementary metrics supports the potential value of multimodal integration beyond AUC comparisons alone. These findings suggest that DL features may complement interpretable radiomic features, generating a promising candidate tool whose potential clinical utility requires validation in external cohorts.

### 4.3 Technical details and methodological innovations

#### 4.3.1 Selection and advantages of machine learning algorithms

Comparing LR, RF, SVM, and XGBoost, this study found that RF most robustly predicted treatment efficacy. By employing bootstrap sampling and random feature selection, RF alleviates overfitting in high-dimensional, small-sample datasets (50–52). Furthermore, RF is tolerant to MRI artifacts (53), enables biomarker identification via intrinsic feature importance (52, 54), and accommodates nonlinear relationships and class imbalance, ultimately improving clinical prediction accuracy (54, 55).

#### 4.3.2 Construction and exploratory interpretation of core features

The 14 features selected for Model-TP6 were analyzed across three dimensions: transformation type, feature class, and spatial location. Regarding transformations, wavelet features constitute 85.71% (12/14) of the total (**Supplementary Table 1**). This high proportion may be attributable to the multiscale spatial-frequency localization capability of the wavelet transform, which enables the extraction of edge and texture information at multiple scales, and is highly sensitive to tumor heterogeneity (56, 57). Zhou et al. (58) also reported in their study that compared to simple volume or edge texture, wavelet-transformed texture features have superior predictive performance for evaluating the efficacy of neoadjuvant therapy in locally advanced breast cancers.

For feature classes, the model included 12 texture features that may characterize imaging heterogeneity within the tumor and its surrounding tissue (20, 21, 59). Spatially, the features comprise 8 intratumoral and 6 peritumoral features (**Figure 6**). Specifically, a highly weighted intratumoral feature (Wavelet_HLL_Glcm_Imc1_T) was negatively associated with pCR, which may reflect more complex internal textures in non-pCR lesions. Similarly, a prominent peritumoral feature (Wavelet_HLH_Glszm_LAHGLE_P6) was positively associated with pCR and may be related to peritumoral microenvironmental heterogeneity. We emphasize, however, that these feature-level interpretations are hypothesis-generating and exploratory; the correspondence between high-dimensional radiomic features and specific biological processes was not directly validated in this study and requires confirmation through dedicated radiopathological or radiogenomic correlation analyses.

#### 4.3.3 Innovative application of 3D deep learning-based features

Most previous studies employed 2D slice-based analysis (24, 60), which may not fully capture through-plane information. However, in breast cancer MRI, three-dimensional intratumoral heterogeneity may influence heterogeneous spatial pattern expression. This study utilized 3D ResNet-50 for deep feature extraction. By preserving inter-slice continuity and spatial context, the 3D approach may capture volumetric heterogeneity that is incompletely represented in 2D slice-based analyses (e.g., spiculation, central necrosis, and angiogenesis).

### 4.4 Limitations and future perspectives

The major limitations of this study are its retrospective, single-center design and the absence of independent external validation. Although the internal test set was strictly held out from all feature selection and model training procedures, both subsets were derived from the same institutional cohort, and all MRI scans were acquired using the same 3.0 T GE Signa HDxt scanner under a uniform acquisition protocol. Consequently, the reported estimates characterize performance within the source institution only and may be optimistic in external settings; the model’s transportability across institutions, MRI vendors, acquisition protocols, and patient populations remains unestablished. Several methodological limitations also remain. The entire radiomics workflow, including ROI segmentation and feature extraction, still lacks standardized protocols, which may introduce technical variability. In addition, the radiomics and deep-learning scores used to fit the fusion model were generated in-sample rather than by out-of-fold prediction. This may have introduced some optimism into the training-set fit. However, the held-out test-set evaluation is unaffected, because the test-set scores are generated out of sample with respect to the base models. Furthermore, the correspondence between high-dimensional features and the underlying biological characteristics of the tumor remains elusive, and the “black-box” nature of deep learning impedes clinicians’ trust in and interpretation of the prediction results. Finally, several clinical limitations should be noted. Reliance on a single MRI sequence limits comprehensive characterization of the multidimensional, cross-scale complex biological behaviors of the tumor; in addition, as only mass-type lesions with delineable margins were analyzed, the findings may not generalize to non-mass-enhancement or ill-defined tumors. Importantly, detailed patient-level regimen information, including specific drug combinations, dose modifications for tolerability, and treatment duration, was not systematically recorded in this retrospective cohort and could therefore not be incorporated as covariates or evaluated in sensitivity analyses. Although treatment allocation generally followed molecular subtype and receptor status, and was thus partially captured by the model’s included receptor variables (ER, PR, HER2, and Ki-67), residual confounding due to within-subtype treatment heterogeneity, which may have influenced pCR, cannot be excluded.

Future research may focus on several strategies to improve the accuracy and clinical utility of neoadjuvant therapy response prediction in breast cancer. To address the generalizability gap identified above, we plan to seek multicenter collaboration with institutions employing different MRI platforms (e.g., Siemens, Philips) to prospectively evaluate model transportability, and we intend to release the feature extraction pipeline to facilitate independent external evaluation. Beyond external validation, future efforts may also adopt out-of-fold stacking to fit the fusion model more rigorously, prospectively collect structured treatment-regimen data to disentangle therapy effects from tumor biology, incorporate visualization techniques to enhance model interpretability, integrate multiparametric MRI with pathological slides and multi-omics data to construct more robust models (61–68), and apply self-supervised and semi-supervised learning (69–71) or federated learning (72) to extract generalizable features across privacy-preserving multicenter settings. Ultimately, once externally validated, such tools could potentially support clinical decision-making throughout the treatment course.

## 5 Conclusion

This study provides preliminary, hypothesis-generating evidence that the 6-mm peritumoral zone may offer incremental predictive value for pCR following breast cancer NST. Notably, the pairwise DeLong comparison between Model-TP6 and the intratumoral-only model did not reach statistical significance (P = 0.358), indicating that the observed advantage represents a numerical trend rather than a statistically confirmed finding. This trend, consistent across all four classifiers, warrants confirmation in larger, adequately powered, and independent external cohorts. Our multimodal nomogram, integrating intra-/peritumoral radiomics, deep-learning signatures, and clinical biomarkers, achieved a test-set AUC of 0.883 (95% CI: 0.827–0.940). Although it significantly outperformed the clinical model (P = 0.005) and Model-TP9 (P = 0.021), its pairwise comparisons with the remaining models did not reach statistical significance. Nevertheless, the nomogram demonstrated a favorable combination of discrimination, calibration (Hosmer–Lemeshow P = 0.115), and decision curve net benefit. The retrospective, single-center, single-scanner design and absence of independent external validation represent major limitations that may have led to optimistic performance estimates and may limit model transportability. The nomogram should therefore be considered exploratory and requires prospective multicenter external validation using a fully prespecified modeling pipeline before clinical deployment. If confirmed, this framework may assist in stratifying NST candidates and informing individualized surgical planning.

## Data Availability

The raw data supporting the conclusions of this article will be made available by the authors, without undue reservation.

## Glossary

NST: Neoadjuvant systemic therapy
pCR: Pathological complete response
DCE-MRI: Dynamic contrast-enhanced magnetic resonance imaging
T1WI: T1-weighted imaging
Gd-DTPA: Gadolinium-diethylenetriamine pentaacetic acid
PACS: Picture archiving and communication system
DICOM: Digital Imaging and Communications in Medicine
TR: Repetition time
TE: Echo time
FOV: Field of view
TME: Tumor microenvironment
ROI: Region of interest
VOI: Volume of interest
DSC: Dice similarity coefficient
ICC: Intraclass correlation coefficient
T: Intratumoral feature set
TP3: Combined intratumoral and 3-mm peritumoral feature set
TP6: Combined intratumoral and 6-mm peritumoral feature set
TP9: Combined intratumoral and 9-mm peritumoral feature set
GLCM: Gray level co-occurrence matrix
GLRLM: Gray level run length matrix
GLSZM: Gray level size zone matrix
GLDM: Gray level dependence matrix
NGTDM: Neighborhood gray tone difference matrix
LoG: Laplacian of Gaussian
DL: Deep learning
SE: Squeeze-and-Excitation
SGD: Stochastic gradient descent
PCA: Principal component analysis
LASSO: Least absolute shrinkage and selection operator
SMOTE: Synthetic minority oversampling technique
LR: Logistic regression
RF: Random forest
SVM: Support vector machine
XGBoost: Extreme gradient boosting
CM: Clinical model
CIPRM: Clinical and intra-peritumoral radiomics model
AUC: Area under the curve
ROC: Receiver operating characteristic
DCA: Decision curve analysis
H-L: Hosmer–Lemeshow
CI: Confidence interval
Acc: Accuracy
Sen: Sensitivity
Spe: Specificity
RQS: Radiomics Quality Score
ER: Estrogen receptor
PR: Progesterone receptor
HER2: Human epidermal growth factor receptor 2
Ki-67: Marker of proliferation Ki-67
IHC: Immunohistochemistry
FISH: Fluorescence *in situ* hybridization
TNM: Tumor–node–metastasis (staging)
BMI: Body mass index

## References

[1] Siegel RL Kratzer TB Giaquinto AN Sung H Jemal A . Cancer statistics, 2025. CA Cancer J Clin. (2025) 75:11–45. doi: 10.3322/caac.21871 39817679 PMC11745215

[2] Spring LM Fell G Arfe A Sharma C Greenup R Reynolds KL . Pathologic complete response after neoadjuvant chemotherapy and impact on breast cancer recurrence and survival: a comprehensive meta-analysis. Clin Cancer Res. (2020) 26:2838–48. doi: 10.1158/1078-0432.CCR-19-3492 32046998 PMC7299787

[3] Jackisch C Hegg R Stroyakovskiy D Ahn J-S Melichar B Chen S-C . Hannah phase III randomised study: Association of total pathological complete response with event-free survival in HER2-positive early breast cancer treated with neoadjuvant–adjuvant trastuzumab after 2 years of treatment-free follow-up. Eur J Cancer. (2016) 62:62–75. doi: 10.1016/j.ejca.2016.03.087 27208905

[4] Cortazar P Zhang L Untch M Mehta K Costantino JP Wolmark N . Pathological complete response and long-term clinical benefit in breast cancer: the CTNeoBC pooled analysis. Lancet. (2014) 384:164–72. doi: 10.1016/S0140-6736(13)62422-8 24529560

[5] Lambin P Rios-Velazquez E Leijenaar R Carvalho S van Stiphout RGPM Granton P . Radiomics: Extracting more information from medical images using advanced feature analysis. Eur J Cancer. (2012) 48:441–6. doi: 10.1016/j.ejca.2011.11.036 22257792 PMC4533986

[6] Qi Y-J Su G-H You C Zhang X Xiao Y Jiang Y-Z . Radiomics in breast cancer: current advances and future directions. Cell Rep Med. (2024) 5:101719. doi: 10.1016/j.xcrm.2024.101719 39293402 PMC11528234

[7] Daimiel Naranjo I Gibbs P Reiner JS Lo Gullo R Sooknanan C Thakur SB . Radiomics and machine learning with multiparametric breast MRI for improved diagnostic accuracy in breast cancer diagnosis. Diagnostics. (2021) 11:919. doi: 10.3390/diagnostics11060919 34063774 PMC8223779

[8] Zhang R Wei W Li R Li J Zhou Z Ma M . An MRI-based radiomics model for predicting the benignity and Malignancy of BI-RADS 4 breast lesions. Front Oncol. (2022) 11:733260. doi: 10.3389/fonc.2021.733260 35155178 PMC8833233

[9] Zhang J Zhan C Zhang C Song Y Yan X Guo Y . Fully automatic classification of breast lesions on multi-parameter MRI using a radiomics model with minimal number of stable, interpretable features. Radiol Med. (2023) 128:160–70. doi: 10.1007/s11547-023-01594-w 36670236

[10] Li H Zhu Y Burnside ES Huang E Drukker K Hoadley KA . Quantitative MRI radiomics in the prediction of molecular classifications of breast cancer subtypes in the TCGA/TCIA data set. NPJ Breast Cancer. (2016) 2:16012. doi: 10.1038/npjbcancer.2016.12 27853751 PMC5108580

[11] Leithner D Horvat JV Marino MA Bernard-Davila B Jochelson MS Ochoa-Albiztegui RE . Radiomic signatures with contrast-enhanced magnetic resonance imaging for the assessment of breast cancer receptor status and molecular subtypes: initial results. Breast Cancer Res. (2019) 21:106. doi: 10.1186/s13058-019-1187-z 31514736 PMC6739929

[12] Han L Zhu Y Liu Z Yu T He C Jiang W . Radiomic nomogram for prediction of axillary lymph node metastasis in breast cancer. Eur Radiol. (2019) 29:3820–9. doi: 10.1007/s00330-018-5981-2 30701328

[13] Chen Y Li J Zhang J Yu Z Jiang H . Radiomic nomogram for predicting axillary lymph node metastasis in patients with breast cancer. Acad Radiol. (2024) 31:788–99. doi: 10.1016/j.acra.2023.10.026 37932165

[14] Yu Y Tan Y Xie C Hu Q Ouyang J Chen Y . Development and validation of a preoperative magnetic resonance imaging radiomics–based signature to predict axillary lymph node metastasis and disease-free survival in patients with early-stage breast cancer. JAMA Netw Open. (2020) 3:e2028086. doi: 10.1001/jamanetworkopen.2020.28086 33289845 PMC7724560

[15] Guo L Du S Gao S Zhao R Huang G Jin F . Delta-radiomics based on dynamic contrast-enhanced MRI predicts pathologic complete response in breast cancer patients treated with neoadjuvant chemotherapy. Cancers. (2022) 14:3515. doi: 10.3390/cancers14143515 35884576 PMC9316501

[16] Shi Z Huang X Cheng Z Xu Z . MRI-based quantification of intratumoral heterogeneity for predicting treatment response to neoadjuvant chemotherapy in breast cancer. Radiology. (2023) 308:e222830. doi: 10.1148/radiol.222830 37432083

[17] Hoffmann E Masthoff M Kunz WG Seidensticker M Bobe S Gerwing M . Multiparametric MRI for characterization of the tumour microenvironment. Nat Rev Clin Oncol. (2024) 21:428–48. doi: 10.1038/s41571-024-00891-1 38641651

[18] Wu J Li B Sun X Cao G Rubin DL Napel S . Heterogeneous enhancement patterns of tumor-adjacent parenchyma at MR imaging are associated with dysregulated signaling pathways and poor survival in breast cancer. Radiology. (2017) 285:401–13. doi: 10.1148/radiol.2017162823 28708462 PMC5673053

[19] Li C Lu N He Z Tan Y Liu Y Chen Y . A noninvasive tool based on magnetic resonance imaging radiomics for the preoperative prediction of pathological complete response to neoadjuvant chemotherapy in breast cancer. Ann Surg Oncol. (2022) 29:7685–93. doi: 10.1245/s10434-022-12034-w 35773561 PMC9550709

[20] Braman NM Etesami M Prasanna P Dubchuk C Gilmore H Tiwari P . Intratumoral and peritumoral radiomics for the pretreatment prediction of pathological complete response to neoadjuvant chemotherapy based on breast DCE-MRI. Breast Cancer Res. (2017) 19:57. doi: 10.1186/s13058-017-0846-1 28521821 PMC5437672

[21] Braman N Prasanna P Whitney J Singh S Beig N Etesami M . Association of peritumoral radiomics with tumor biology and pathologic response to preoperative targeted therapy for *HER2 (ERBB2)*–positive breast cancer. JAMA Netw Open. (2019) 2:e192561. doi: 10.1001/jamanetworkopen.2019.2561 31002322 PMC6481453

[22] G S Meenakshisundaram N . (2025). “ An improved diagnostic process to identify breast cancer in earlier stages using deep learning mechanism”, in: 2025 8th International Conference on Trends in Electronics and Informatics (ICOEI) (Tirunelveli, India: IEEE), 1637–42. doi: 10.1109/ICOEI65986.2025.11013687

[23] Meenakshisundaram N G S . (2025). “ OCNC: efficient learning mechanism to identify breast cancer disease using optimized convolutional neural classification scheme”, in: 2025 7th International Conference on Signal Processing, Computing and Control (ISPCC) (Solan, India: IEEE), 727–32. doi: 10.1109/ISPCC66872.2025.11039384

[24] Qu Y-H Zhu H-T Cao K Li X-T Ye M Sun Y-S . Prediction of pathological complete response to neoadjuvant chemotherapy in breast cancer using a deep learning (DL) method. Thorac Cancer. (2020) 11:651–8. doi: 10.1111/1759-7714.13309 31944571 PMC7049483

[25] Zhou Z Adrada BE Candelaria RP Elshafeey NA Boge M Mohamed RM . Prediction of pathologic complete response to neoadjuvant systemic therapy in triple negative breast cancer using deep learning on multiparametric MRI. Sci Rep. (2023) 13:1171. doi: 10.1038/s41598-023-27518-2 36670144 PMC9859781

[26] Kim S-Y Lee J Cho N Kim Y-G . Deep-learning based discrimination of pathologic complete response using MRI in HER2-positive and triple-negative breast cancer. Sci Rep. (2024) 14:23065. doi: 10.1038/s41598-024-74276-w 39367159 PMC11452398

[27] Chen Z-H Lin L Wu C-F Li C-F Xu R-H Sun Y . Artificial intelligence for assisting cancer diagnosis and treatment in the era of precision medicine. Cancer Commun. (2021) 41:1100–15. doi: 10.1002/cac2.12215 34613667 PMC8626610

[28] van der Velden BHM Kuijf HJ Gilhuijs KGA Viergever MA . Explainable artificial intelligence (XAI) in deep learning-based medical image analysis. Med Image Anal. (2022) 79:102470. doi: 10.1016/j.media.2022.102470 35576821

[29] Hammond MEH Hayes DF Dowsett M Allred DC Hagerty KL Badve S . American society of clinical oncology/college of American pathologists guideline recommendations for immunohistochemical testing of estrogen and progesterone receptors in breast cancer. J Clin Oncol. (2010) 28:2784–95. doi: 10.1200/JCO.2009.25.6529 20404251 PMC2881855

[30] Wolff AC Hammond MEH Hicks DG Dowsett M McShane LM Allison KH . Recommendations for human epidermal growth factor receptor 2 testing in breast cancer: american society of clinical oncology/college of American pathologists clinical practice guideline update. J Clin Oncol. (2013) 31:3997–4013. doi: 10.1200/JCO.2013.50.9984 24101045

[31] Korde LA Somerfield MR Carey LA Crews JR Denduluri N Hwang ES . Neoadjuvant chemotherapy, endocrine therapy, and targeted therapy for breast cancer: ASCO guideline. J Clin Oncol. (2021) 39:1485–505. doi: 10.1200/JCO.20.03399 33507815 PMC8274745

[32] Liu Z Wang S Dong D Wei J Fang C Zhou X . The applications of radiomics in precision diagnosis and treatment of oncology: opportunities and challenges. Theranostics. (2019) 9:1303–22. doi: 10.7150/thno.30309 30867832 PMC6401507

[33] Satake H Ishigaki S Ito R Naganawa S . Radiomics in breast MRI: current progress toward clinical application in the era of artificial intelligence. Radiol Med. (2022) 127:39–56. doi: 10.1007/s11547-021-01423-y 34704213

[34] Conti A Duggento A Indovina I Guerrisi M Toschi N . Radiomics in breast cancer classification and prediction. Semin Cancer Biol. (2021) 72:238–50. doi: 10.1016/j.semcancer.2020.04.002 32371013

[35] Zhang X Zhang Y Zhang G Qiu X Tan W Yin X . Deep learning with radiomics for disease diagnosis and treatment: challenges and potential. Front Oncol. (2022) 12:773840. doi: 10.3389/fonc.2022.773840 35251962 PMC8891653

[36] Nielsen TO Leung SCY Rimm DL Dodson A Acs B Badve S . Assessment of Ki67 in breast cancer: updated recommendations from the international Ki67 in breast cancer working group. JNCI: J Natl Cancer Inst. (2021) 113:808–19. doi: 10.1093/jnci/djaa201 33369635 PMC8487652

[37] Toli MA Liu X Massa D Lando S Boman C Tsiknakis N . Prognostic significance of tumour ki-67 dynamics during neoadjuvant treatment in patients with breast cancer: a population-based cohort study. Lancet Reg Health Eur. (2025) 58:101432. doi: 10.1016/j.lanepe.2025.101432 40980814 PMC12444493

[38] Li X Cheng H Zhao C Tu G . Predictive values of clinicopathological parameters and molecular typing for pathological complete response (pCR) in breast cancer neoadjuvant chemotherapy by TEC. Cancer Res Prev Treat. (2013) 40:599–603. doi: 10.3971/j.issn.1000-8578.2013.06.023

[39] Akdag G Yildirim S Dogan A Yuksel Yasar Z Bal H Kinikoglu O . Neoadjuvant chemotherapy and pathologic complete response in HR+/HER2- breast cancer: impact of tumor Ki67 and ER status. Chemotherapy. (2024) 69:141–9. doi: 10.1159/000537874 38368871

[40] Olapo JS Brennan SK Javid SH Flanagan MR . Neoadjuvant systemic therapy versus up-front surgery in ER +/PR +/HER2- breast cancer. Curr Breast Cancer Rep. (2025) 17:19. doi: 10.1007/s12609-025-00578-130311153

[41] Fan Y Wang Y He L Imani S Wen Q . Clinical features of patients with HER2-positive breast cancer and development of a nomogram for predicting survival. ESMO Open. (2021) 6:100232. doi: 10.1016/j.esmoop.2021.100232 34392135 PMC8371219

[42] Bae SJ Kim JH Lee MJ Baek SH Kook Y Ahn SG . Predictive markers of treatment response to neoadjuvant systemic therapy with dual HER2-blockade. Cancers. (2024) 16:842. doi: 10.3390/cancers16040842 38398233 PMC10886516

[43] Pu S Wang K Liu Y Liao X Chen H He J . Nomogram-derived prediction of pathologic complete response (pCR) in breast cancer patients treated with neoadjuvant chemotherapy (NCT). BMC Cancer. (2020) 20:1120. doi: 10.1186/s12885-020-07621-7 33213397 PMC7678042

[44] Xie T Gong J Zhao Q Wu C Wu S Peng W . Development and validation of peritumoral vascular and intratumoral radiomics to predict pathologic complete responses to neoadjuvant chemotherapy in patients with triple-negative breast cancer. BMC Med Imaging. (2024) 24:136. doi: 10.1186/s12880-024-01311-7 38844842 PMC11155097

[45] Tu Y Weng A Ren A Dong P . Multimodal MRI manifestations and pathological basis of peritumoral infiltration of glioma in rat. Chin J Magn Reson Imaging. (2023) 14:105–10. doi: 10.12015/issn.1674-8034.2023.01.019

[46] Zhou J Yu X Wu Q Wu Y Fu C Wang Y . Radiomics analysis of intratumoral and different peritumoral regions from multiparametric MRI for evaluating HER2 status of breast cancer: a comparative study. Heliyon. (2024) 10:e28722. doi: 10.1016/j.heliyon.2024.e28722 38623231 PMC11016612

[47] ZhengW SunZ WangW HanJE IslamMT LiW . Noninvasive imaging-based assessment of tumor-associated neutrophils for prognosis and immunotherapy response in gastric cancer: a multicenter study. J Adv Res. (2026). doi: 10.1016/j.jare.2026.01.08241633486

[48] Li J Lv D Guo Z Zhou H Yao X Rong Y . Integration of multi-scale radiomics and deep learning for ki-67 prediction in clear cell renal carcinoma. NPJ Precis Oncol. (2026) 10:12. doi: 10.1038/s41698-025-01214-y 41353523 PMC12783816

[49] Peng Y Cheng Z Gong C Zheng C Zhang X Wu Z . Pretreatment DCE-MRI-based deep learning outperforms radiomics analysis in predicting pathologic complete response to neoadjuvant chemotherapy in breast cancer. Front Oncol. (2022) 12:846775. doi: 10.3389/fonc.2022.846775 35359387 PMC8960929

[50] Wang X-H Long L-H Cui Y Jia AY Zhu X-G Wang H-Z . MRI-based radiomics model for preoperative prediction of 5-year survival in patients with hepatocellular carcinoma. Br J Cancer. (2020) 122:978–85. doi: 10.1038/s41416-019-0706-0 31937925 PMC7109104

[51] Ingrisch M Schneider M Nörenberg D Negrao De Figueiredo G Maier-Hein K Suchorska B . Radiomic analysis reveals prognostic information in T1-weighted baseline magnetic resonance imaging in patients with glioblastoma. Invest Radiol. (2017) 52:361–6. doi: 10.1097/RLI.0000000000000349 28079702

[52] Ravikiran H Deepak R Deepak H Prapulla Kumar M Sharath S Yogeesh G . A robust framework for alzheimer’s disease detection and staging: incorporating multi-feature integration, MRMR feature selection, and random forest classification. Multimedia Tools Appl. (2024) 84:24903–31. doi: 10.1007/s11042-024-19875-530311153

[53] Perniciano A Loddo A Di Ruberto C Pes B . Insights into radiomics: impact of feature selection and classification. Multimedia Tools Appl. (2024) 84:31695–721. doi: 10.1007/s11042-024-20388-430311153

[54] Lee J Lee K Seo B Cho K Woo O Song S . Radiomic machine learning for predicting prognostic biomarkers and molecular subtypes of breast cancer using tumor heterogeneity and angiogenesis properties on MRI. Eur Radiol. (2022) 32:650–60. doi: 10.1007/s00330-021-08146-8 34226990

[55] Liu D Zhang X Zheng T Shi Q Cui Y Wang Y . Optimisation and evaluation of the random forest model in the efficacy prediction of chemoradiotherapy for advanced cervical cancer based on radiomics signature from high-resolution T2 weighted images. Arch Gynecol Obstet. (2021) 303:811–20. doi: 10.1007/s00404-020-05908-5 33394142 PMC7960581

[56] Shuvo S Alam S Ayman S Chakma A Salvi M Seoni S . Application of wavelet transformation and artificial intelligence techniques in healthcare: a systemic review. WIREs Data Min Knowl Discov. (2025) 15:e70007. doi: 10.1002/widm.7000742587375

[57] Tang V Duong S Nguyen C Huynh T Duc V Phan C . Wavelet radiomics features from multiphase CT images for screening hepatocellular carcinoma: Analysis and comparison. Sci Rep. (2023) 13:19559. doi: 10.1038/s41598-023-46695-8 37950031 PMC10638447

[58] Zhou J Lu J Gao C Zeng J Zhou C Lai X . Predicting the response to neoadjuvant chemotherapy for breast cancer: Wavelet transforming radiomics in MRI. BMC Cancer. (2020) 20:100. doi: 10.1186/s12885-020-6523-2 32024483 PMC7003343

[59] Park J Kim M Yoon J-H Han K Kim E-K Sohn J . Machine learning predicts pathologic complete response to neoadjuvant chemotherapy for ER+HER2- breast cancer: integrating tumoral and peritumoral MRI radiomic features. Diagnostics. (2023) 13:3031. doi: 10.3390/diagnostics13193031 37835774 PMC10572844

[60] Li X Yang L Jiao X . Comparison of traditional radiomics, deep learning radiomics and fusion methods for axillary lymph node metastasis prediction in breast cancer. Acad Radiol. (2023) 30:1281–7. doi: 10.1016/j.acra.2022.10.015 36376154

[61] Du Y Zhu Y Yang M Zhang A Zhang L Zhou Z . Deep learning model based on DCE-MRI: fusion of 3D features of tumor, peritumoral vessels and metastatic lymph nodes for prediction of pathological complete response to neoadjuvant therapy in breast cancer. Front Oncol. (2025) 15:1664631. doi: 10.3389/fonc.2025.1664631 41409238 PMC12705360

[62] Xu S Ying Y Hu Q Li X Li Y Xiong H . Fusion model integrating multi-sequence MRI radiomics and habitat imaging for predicting pathological complete response in breast cancer treated with neoadjuvant therapy. Cancer Imaging. (2025) 25:108. doi: 10.1186/s40644-025-00929-2 40883826 PMC12395702

[63] Yuan Q Hong Z Ye R Yang P Lin J Jiang X . Integrating deep feature extraction and MRI radiomics for survival prediction in breast cancer after neoadjuvant chemotherapy. Acad Radiol. (2026) 33:872–88. doi: 10.1016/j.acra.2025.10.050 41253608

[64] Liu J Wang X Mao N Hua H Zhong X Han J . The value of machine learning based on magnetic resonance imaging (MRI) and biopsy whole-slide image to predict pathological complete response to breast cancer after neoadjuvant chemotherapy: a two-centre study. Clin Radiol. (2025) 87:106976. doi: 10.1016/j.crad.2025.106976 40582270

[65] Xu N Guo X Ouyang Z Ran F Li Q Duan X . Multiparametric MRI-based radiomics combined with pathomics features for prediction of the efficacy of neoadjuvant chemotherapy in breast cancer. Heliyon. (2024) 10:e24371. doi: 10.1016/j.heliyon.2024.e24371 38298695 PMC10827766

[66] Duan J Zhao Y Sun Q Liang D Liu Z Chen X . Imaging-proteomic analysis for prediction of neoadjuvant chemotherapy responses in patients with breast cancer. Cancer Med. (2023) 12:21256–69. doi: 10.1002/cam4.6704 37962087 PMC10726892

[67] Zhang T Zhao L Cui T Zhou Y Li P Luo C . Spatial-temporal radiogenomics in predicting neoadjuvant chemotherapy efficacy for breast cancer: a comprehensive review. J Transl Med. (2025) 23:681. doi: 10.1186/s12967-025-06641-w 40533825 PMC12178003

[68] Huang Y Zhu T Zhang X Li W Zheng X Cheng M . Longitudinal MRI-based fusion novel model predicts pathological complete response in breast cancer treated with neoadjuvant chemotherapy: a multicenter, retrospective study. eClinicalMedicine. (2023) 58:101899. doi: 10.1016/j.eclinm.2023.101899 37007742 PMC10050775

[69] Huang X Xu Z Zhao Y Wang Y Liu Y Hu W . Longitudinal MRI-based deep learning model for predicting pathological complete response in breast cancer: a multicenter, retrospective cohort study. NPJ Precis Oncol. (2026) 10:57. doi: 10.1038/s41698-025-01256-2 41491286 PMC12886838

[70] Huang S-C Pareek A Jensen M Lungren M Yeung S Chaudhari A . Self-supervised learning for medical image classification: a systematic review and implementation guidelines. NPJ Digit Med. (2023) 6:74. doi: 10.1038/s41746-023-00811-0 37100953 PMC10131505

[71] Hao X Xu H Zhao N Yu T Hamalainen T Cong F . Predicting pathological complete response based on weakly and semi-supervised joint learning in breast cancer multi-parametric MRI. BioMed Signal Process Control. (2024) 93:106164. doi: 10.1016/j.bspc.2024.10616442574925

[72] Hanser T . Federated learning for molecular discovery. Curr Opin Struct Biol. (2023) 79:102545. doi: 10.1016/j.sbi.2023.102545 36804704

