# Supplementary material for: A radiomics–deep learning nomogram integrating intratumoral and peritumoral DCE-MRI features for pCR prediction in breast cancer
Source: Front Oncol. 2026 Sep 16;16:1830219. doi: 10.3389/fonc.2026.1830219 (PMC13623723; doi:10.3389/fonc.2026.1830219)
Supplement: Supplementary file 1 [file DataSheet1.pdf]

Supplementary Material

1.1 Supplementary Figures

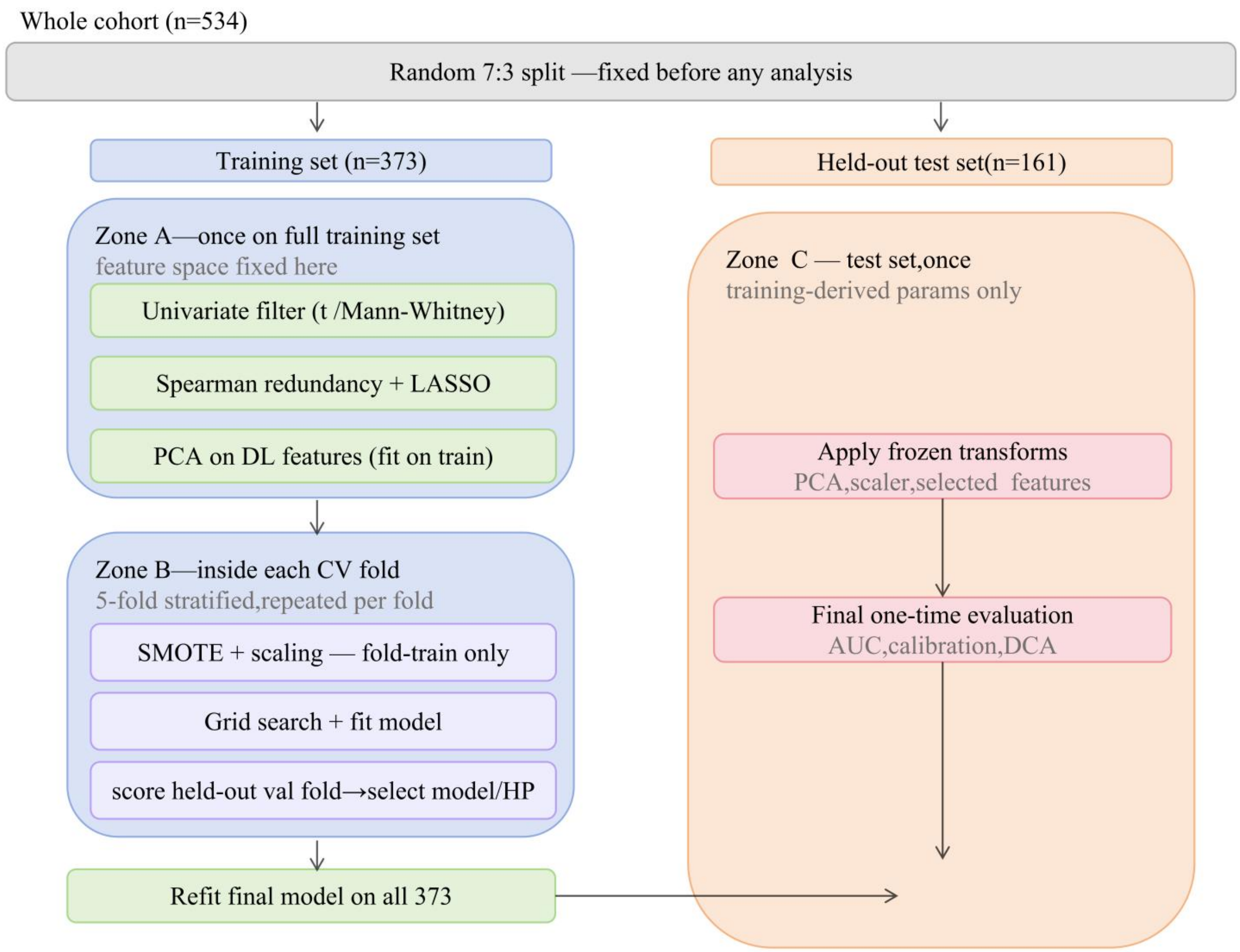

Supplementary Figure S1 Modeling pipeline with strict held-out test-set separation.Test set never enters Zone A or Zone B — no role in selection or tuning; CV (Zone B) selects model/hyperparameters; final performance is reported on the test set only.

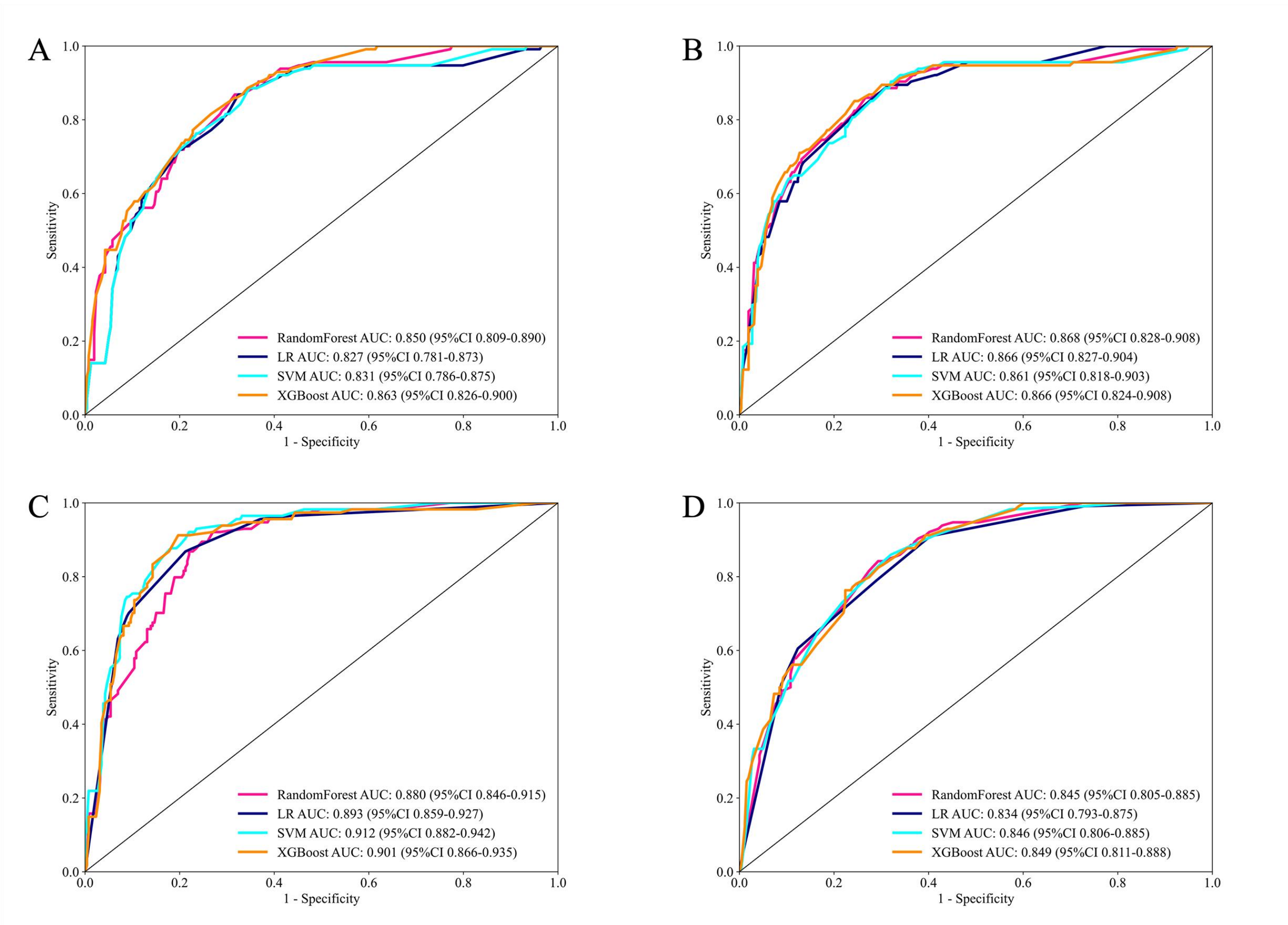

Supplementary Figure S2 Comparison of ROC curves of machine learning models based on feature sets from different regions in the training set.

1.2 Supplementary Tables

Supplementary Table S1 Radiomics features with non-zero coefficients retained by LASSO at the selected λ and their corresponding coefficients.

Model	Image filters	Feature type	Features	Coefficient
Tumor	Log sigma 2.0mm 3D	Glcm	Correlation	-0.006
	wavelet-HHH	Glcm	Correlation	+0.029
	wavelet-HHH	Glcm	Imc2	+0.015
	wavelet-HHH	Gldm	Large Dependence Low Gray Level Emphasis	-0.010
	wavelet-HHH	Glszm	Zone Entropy	+0.019
	wavelet-HHL	Glszm	Large Area Low Gray Level Emphasis	+0.021
	wavelet-HLH	First order	Median	-0.043
	wavelet-HLH	Glcm	Correlation	+0.031
	wavelet-HLL	Glcm	Cluster Prominence	-0.008
	wavelet-HLL	Glcm	Imc1	-0.045
	wavelet-LHH	Glszm	Small Area Emphasis	+0.013
	wavelet-LHL	Ngtdm	Busyness	+0.010
	wavelet-LLL	Glcm	Correlation	-0.023
	wavelet-LLL	Glszm	Zone Variance	-0.001
TP3	Log sigma 2.0mm 3D	Glcm	Correlation	-0.004
	Wavelet-HHH	Glcm	Correlation	+0.024
	Wavelet-HHH	Glcm	Imc2	+0.018
	Wavelet-HHH	Gldm	Large Dependence Low Gray Level Emphasis	-0.002
	Wavelet-HHH	Glszm_	Zone Entropy	+0.002
	Wavelet-HHL	Glszm	Small Area Emphasis	+0.005
	Wavelet-HHL	Glszm	Zone Variance	+0.012
	Wavelet-HLH	First order	Median	-0.024
	Wavelet-HLH	Glcm	Correlation	+0.004
	Wavelet-HLL	Glcm	Imc1	-0.034
	Wavelet-LHH	Glszm	Small Area Emphasis	+0.010
	Wavelet-LLL	Glcm	Correlation	-0.011
	Wavelet-HHL	Ngtdm	Strength	+0.001
	Wavelet-HLH	Glcm	Correlation	+0.017
TP6	Log sigma 2.0mm 3D	Glcm	Correlation	-0.002
	Wavelet-HHH	Glcm	Correlation	+0.025
	Wavelet-HHH	Glcm	Imc2	+0.017
	Wavelet-HLH	First order	Median	-0.026
	Wavelet-HLH	Glcm	Correlation	+0.016
	Wavelet-HLL	Glcm	Imc1	-0.031
	Wavelet-LHH	Glszm	Small Area Emphasis	+0.006
	Wavelet-LLL	Glcm	Correlation	-0.013
	Log sigma 3.0mm 3D	Glcm	Imc1	+0.001

Model	Image filters	Feature type	Features	Coefficient
TP9	Wavelet-HHH	Glrlm	Short Run Emphasis	+0.002
	Wavelet-HHL	Ngtdm	Strength	+0.007
	Wavelet-HLH	Glszm	Large Area High Gray Level Emphasis	+0.016
	Wavelet-LHL	Glszm	Zone Percentage	+0.006
	Wavelet-LLL	First order	Median	+0.0001
	Log sigma 2.0mm 3D	Glcm	Correlation	-0.002
	Wavelet-HHH	Glcm	Correlation	+0.030
	Wavelet-HHH	Glcm	Imc2	+0.021
	Wavelet-HHL	Glszm	Zone Variance	+0.019
	Wavelet-HLH	First order	Median	-0.034
	Wavelet-HLH	Glcm	Correlation	+0.031
	Wavelet-HLL	Glcm	Imc1	-0.025
	Wavelet-LLL	Glcm	Correlation	-0.013
	Original	Glrlm	Run Variance	-0.0004
	Original	Shape	Flatness	-0.020
	Wavelet-HHH	Gldm	DependenceVariance	-0.035
	Wavelet-HHL	Gldm	Large Dependence Low Gray Level Emphasis	-0.002
	Wavelet-LLL	First order	Median	+0.002
DL			DL 144	-0.004
			DL 183	+0.010
			DL 262	-0.002
			DL 279	+0.001
			DL 343	+0.027
			DL 391	-0.015
			DL 497	+0.014

Supplementary Table S2 Comparison of predictive performance of machine learning models based on feature sets from different regions in the training set.

Regional model	Classifier	AUC	95%CI	Acc	Sen	Spe	F1
Model-T	RF	0.850	0.809-0.890	0.740	0.868	0.683	0.671
	LR	0.827	0.781-0.873	0.735	0.868	0.676	0.667
	SVM	0.831	0.786-0.875	0.724	0.886	0.653	0.662
	XG Boost	0.863	0.826-0.900	0.759	0.816	0.734	0.674
Model-TP3	RF	0.868	0.828-0.908	0.772	0.860	0.734	0.698
	LR	0.866	0.827-0.904	0.748	0.895	0.683	0.685
	SVM	0.861	0.818-0.903	0.748	0.904	0.680	0.687
	XG Boost	0.866	0.824-0.908	0.786	0.851	0.757	0.708
Model-TP6	RF	0.880	0.846-0.915	0.788	0.921	0.730	0.727
	LR	0.893	0.859-0.927	0.812	0.868	0.788	0.739
	SVM	0.912	0.882-0.942	0.823	0.921	0.780	0.761
	XG Boost	0.901	0.867-0.935	0.836	0.912	0.803	0.773
Model-TP9	RF	0.845	0.805-0.885	0.748	0.842	0.707	0.671
	LR	0.834	0.793-0.875	0.694	0.904	0.602	0.644
	SVM	0.846	0.806-0.885	0.735	0.860	0.680	0.664
	XG Boost	0.849	0.811-0.888	0.772	0.763	0.776	0.672

Supplementary Table S3 Calibration slope, calibration intercept, and Brier score of the combined model in the training and internal test cohorts.

Cohort	Calibration slope	Calibration intercept	Brier score
Train	1.220	0.114	0.073
Test	0.818	0.328	0.133

Supplementary Table S4 Performance metrics with 95% confidence intervals for the combined model in the test set.

Metric	Value	95% CI
Accuracy	0.851	0.788–0.898
Sensitivity	0.815	0.692–0.896
Specificity	0.869	0.792–0.920
PPV	0.759	0.635–0.850
NPV	0.903	0.830–0.946
F1 score	0.786	/

*F1 score is reported as a point estimate; 95% CIs for proportion-based metrics were calculated using the Wilson method.

Supplementary Table S5 Radiomics Quality Score (RQS) assessment of the present study.

No.	RQS criterion	Selection for this study	Score	Where reported
1	Image protocol quality	Protocols well documented	+1	Section 2.2 (3.0 T GE Signa HDxt; coil, contrast dose, TR/TE, slice thickness, FOV, etc.)
2	Multiple segmentations / feature robustness to segmentation	Yes	+1	Section 2.3.2 (independent double-blinded segmentation of 50 cases; Dice >0.85; ICC >0.75 feature-robustness screening)
3	Phantom study on all scanners	No	0	Not performed
4	Imaging at multiple time points	No	0	Single pre-treatment baseline acquisition
5	Feature reduction / adjustment for multiple testing	Either measure implemented	+3	Sections 2.4.2, 2.5 (t-test/Mann-Whitney filtering, Spearman redundancy removal, LASSO; PCA for DL features)
6	Multivariable analysis with non-radiomics features	Yes	+1	Sections 2.6.2-2.6.3 (clinical predictors ER/PR/HER2/Ki-67 integrated with radiomics and DL features)
7	Detect and discuss biological correlates	Yes	+1	Section 4.3.2 (biological interpretation of wavelet texture and peritumoral features)
8	Cut-off analyses	Yes	+1	Section 2.6.4 (Youden-index cut-off determined on training set; continuous risk probability reported)
9	Discrimination statistics	Discrimination statistic with significance reported; resampling also applied	+2	Sections 2.6.4, 3.3 (AUC with 95% CI and DeLong test; 5-fold cross-validation)
10	Calibration statistics	Calibration statistic with significance reported; resampling also applied	+2	Sections 2.6.4, 3.3.2 (calibration curves and Hosmer-Lemeshow test; 5-fold cross-validation)
11	Prospective study registered in trial database	No	0	Retrospective design
12	Validation	Validation based on a dataset from the same institute (held-out internal test set)	+2	Sections 2.1.1, 3 (held-out internal test set, n=161, same institution)
13	Comparison to gold standard	No	0	Not formally compared
14	Potential clinical utility	Yes	+2	Sections 3.3.4, 4.1 (decision curve analysis; defined threshold range and decision context)
15	Cost-effectiveness analysis	No	0	Not performed
16	Open science and data	No	0	Data available from authors on

No.	RQS criterion	Selection for this study	Score	Where reported
				reasonable request
	Total RQS score		16 / 36	

The Radiomics Quality Score (RQS) follows Lambin et al., Nat Rev Clin Oncol 2017. For criterion 12, the held-out internal test set (same institution) corresponds to the "validation based on a dataset from the same institute" category; external/prospective validation was not performed and is identified as an essential next step in the Discussion.
